# Differential and unique patterns of synaptic miRNA expression in dorsolateral prefrontal cortex of depressed subjects

**DOI:** 10.1038/s41386-020-00861-y

**Published:** 2020-09-12

**Authors:** Yuta Yoshino, Bhaskar Roy, Yogesh Dwivedi

**Affiliations:** Department of Psychiatry and Behavioral Neurobiology, University of Alabama at Birmingham, Birmingham, AL 35294 USA

**Keywords:** Depression, Depression

## Abstract

Altered synaptic plasticity is often associated with major depressive disorder (MDD). Disease-associated changes in synaptic functions are tightly correlated with altered microRNA (miRNA) expression. Here, we examined the role of miRNAs and their functioning at the synapse in MDD by examining miRNA processing machinery at synapse and sequencing miRNAs and analyzing their functions in synaptic and total tissue fractions obtained from dorsolateral prefrontal cortex (dlPFC) of 15 MDD and 15 matched non-psychiatric control subjects. A total of 333 miRNAs were reliably detected in the total tissue fraction. Multiple testing following the Benjamini–Hochberg false discovery rate [FDR] showed that 18 miRNAs were significantly altered (1 downregulated 4 up and 13 downregulated; *p* < 0.05) in MDD subjects. Out of 351 miRNAs reliably expressed in the synaptic fraction, 24 were uniquely expressed at synapse. In addition, 8 miRNAs (miR-215-5p, miR-192-5p, miR-202-5p, miR-19b-3p, miR-423-5p, miR-219a-2-3p; miR-511-5p, miR-483-5p showed significant (FDR corrected; *p* < 0.05) differential regulation in the synaptic fraction from dlPFC of MDD subjects. In vitro transfection studies and gene ontology revealed involvement of these altered miRNAs in synaptic plasticity, nervous system development, and neurogenesis. A shift in expression ratios (synaptic vs. total fraction) of miR-19b-3p, miR-376c-3p, miR-455-3p, and miR-337-3p were also noted in the MDD group. Moreover, an inverse relationship between the expression of precursor (pre-miR-19b-1, pre-miR-199a-1 and pre-miR-199a-2) and mature (miR-19b-3p, miR-199a-3p) miRNAs was found. Although not significantly, several miRNA processing enzymes (DROSHA [95%], DICER [17%], TARBP2 [38%]) showed increased expression patterns in MDD subjects. Our findings provide new insights into the understanding of the regulation of miRNAs at the synapse and their possible roles in MDD pathogenesis.

## Introduction

Major depressive disorder (MDD) is one of the most debilitating mental disorders worldwide with a lifetime prevalence of 10.8% [1]. Despite significant effort, the pathophysiology of MDD is not well understood. Some of the most prominent findings in MDD are reduced brain plasticity, loss of synaptic connections, and impaired synaptogenesis [2], which could be the consequence of altered molecular pathways and underlying gene regulatory networks [3]. Recently, microRNAs (miRNAs), members of small noncoding RNA families, have received much attention for their unique ability to control complex gene regulatory networks [4, 5]. Mammalian miRNA biogenesis is a programmed pathway which starts canonically with the transcription of a primary transcript (pri-miRNA) by RNA polymerase II/III in nuclei. Pri-miRNAs are ~1 kb in size and contain typical stem-loop structure. With the help of a microprocessor complex (primarily consisting of DROSHA and DGCR8), pri-miRNAs are cropped into smaller hairpin structures (65nts) called precursor miRNAs (pre-miRNAs). Following canonical biogenesis pathway, pre-miRNAs are exported into the cytosol in an Exportin-5 (XPO5)/Ran-GTP-dependent manner and further processed by RNase III DICER. Finally, mature miRNAs are incorporated into the RNA-induced silencing (RISC) complex, which regulates the expression of target genes via translational blockage, transcript degradation, or deadenylation [5]. miRNAs can quickly respond to environmental changes and may generate a highly regulated gene network(s) that can profoundly impact behavior [6]. Our laboratory has previously shown the role of miRNAs in adaptive and maladaptive response to stressful stimuli, a critical factor in MDD pathogenesis [7]. In addition, the role of miRNAs in synaptic plasticity, under both neurodevelopmental and pathogenic conditions, is well documented [8]. Our postmortem brain studies in MDD subjects have shown network-level changes in miRNAs that can target downstream genes involved in neural plasticity and synaptic functions [9]. Interestingly, a possible role of miRNA processing machinery locally at the synapse has been suggested for activity-dependent changes in miRNA expression. In this regard, it has been shown that not only is miRNA maturational machinery available at the synapse [10], but that miRNA biogenesis can occur in the postsynaptic densities (PSDs) near synapse [10–12]. Also, it has been shown that a subset of miRNAs is expressed at a higher level in synaptic fraction than the whole cell lysate isolated from mouse brain [11], which could be the result of an active mobilization of pre-miRNAs and their cleavage into mature miRNAs near synapse with the help of DICER, TRBP and other processing molecules [10, 13]. Reports also indicate the role of local synaptic activity in triggering the release of certain mRNA from miRNA mediated masking, which might facilitate local protein translation in synaptic projections [10, 13–15]. Although it is a growing area of interest, not much has been studied to understand the role of synaptic miRNAs and their underlying regulation in neuropsychiatric disorders.

In the present study, we determined differential expression patterns and functions of miRNAs in synaptic and total tissue fractions isolated from dorsolateral prefrontal cortex (dlPFC) of MDD subjects and matched healthy controls. We chose dlPFC because of its critical role in MDD pathogenesis [16]. For example, dlPFC receives input from specific sensory cortices and is densely interconnected with premotor areas and is involved in executive and cognitive functions such as intention formation, goal-directed action, and attentional control [17]. Additionally, imaging studies reveal functional role of dlPFC where hypoactive resting state is associated with MDD pathogenesis [18]. dlPFC is also involved in activating hypothalamic-pituitary-adrenal (HPA) axis in response to stress as well as in negative feedback regulation [19, 20]. Synapse related gene expression changes have also been reported in dlPFC of MDD subjects [21]. Next, we examined the enrichment of miRNAs in the synaptic fraction by comparing total and synaptic miRNA expression ratios. Unique miRNA expressions were determined by comparing total and synaptic miRNA expression patterns. In addition, we examined the expression of select pre-miRNAs and corresponding mature miRNAs, miRNA processing machinery, and target genes in the synaptic fraction. In vitro analyses were performed to determine the function of select miRNAs. Our overall findings suggest that in MDD brain, miRNA expression may be locally regulated at synapse that might have a significant impact on downstream gene regulatory network(s) involved in synaptic functions.

## Materials and methods

A detailed methodology is discussed in the accompanying supplemental section. The study comes under exemption 4 and was approved by the Institutional Review Board of the University of Alabama at Birmingham.

### Human postmortem brain studies

#### Subjects

The study was performed in dlPFC (Brodmann’s area 46) of 15 MDD and 15 non-psychiatric control subjects (referred hereafter as controls) obtained from Alabama Brain Collection and Maryland Brain Collection programs. Detailed tissue dissection is provided in the supplemental section. The demographic and clinical characteristics of subjects are shown in Table S1. The psychiatric diagnoses were determined by the method of psychological autopsy as detailed in the supplemental section. After receiving written informed consent, at least one member/informant from the family underwent an interview based on the Diagnostic Evaluation After Death (DEAD) [22] and the Structured Clinical Interview for the DSM-V (SCID) [23]. Both cases and controls were characterized by the same psychological autopsy method. There were no significant differences in age, postmortem interval (PMI), and brain pH between MDD and control subjects (Table S1). MDD group had 7 males and 8 females, whereas control group had 8 males and 7 females. Out of 15 MDD subjects, 6 showed positive antidepressant toxicology, 3 had alcohol abuse, and 1 had a history of drug abuse. None of the control subjects had any history of alcohol or drug abuse and were not taking any antidepressants.

#### Synaptosome preparation and characterization

Synaptosomes were isolated by the modified method of Smalheiser and Collins [24] and Lugli et al. [12]. Briefly, 100 mg tissue was homogenized using pestle (total fraction) and centrifuged at 20,000 × *g* x 20 min at 4 °C. The supernatant (S fraction) and pellet were collected. Afterward, sucrose gradient centrifugation was conducted to obtain purified synaptosomes using the resuspended pellet. Twenty micrograms of protein for each isolated fraction (total, S, and synaptosome) were subjected to SDS-PAGE (sodium dodecyl sulfate based polyacrylamide gel electrophoresis) as described in the supplemental section. PCNA, PSD95, and Synapsin I antibodies were used to validate synaptic fraction preparation as detailed in the supplemental section.

#### Isolation of RNA from total and synaptosome fractions

TRIzol ® (Invitrogen, Grand Island, NY, USA) was used to isolate RNA as described earlier [7]. RNA purity was checked by Nanodrop (260/280 nm; cutoff ≥ 1.8) and their integrity by agarose gel electrophoresis (Fig. S1A).

#### Library construction and sequencing of miRNAs

miRNA based transcriptomic expression in synaptosomes was measured using next-generation sequencing (NGS) platform. Total RNA prepared from purified synaptosomes was used to prepare the miRNA sequencing library for each sample. Briefly, the NGS library was prepared using NEB Multiplex Small RNA Library Prep Set for Illumina (New England Biolabs, Ipswich, MA, USA), which included the following steps: (1) 3′-adapter ligation by T4 RNA ligase 2; (2) 5′-adapter ligation by T4 RNA ligase; (3) cDNA synthesis by reverse transcription; (4) low cycle PCR amplification of the library DNA; and (5) size selection by polyacrylamide gel electrophoresis of 135~155 bp PCR amplified fragments (corresponding to ~15–35nt small RNAs). After the libraries were prepared for each sample, they were quantified with Agilent 2100 Bioanalyzer and their qualities were checked. Next, the DNA fragments in the libraries were denatured with alkaline treatment (0.1 M NaOH) to generate single-stranded DNA molecules, captured on Illumina flow cells, amplified in situ, and finally sequenced for 51 cycles on Illumina NextSeq 500 (Illumina, San Diego, CA, USA) according to the manufacturer’s instruction. Raw sequencing data generated, that passed the Illumina chastity filter, were used for further analysis.

#### Bio-computational analysis of miRNA sequencing data

The raw sequencing reads were removed from the adapter sequence as the trimmed reads by cutadapt software. Reads ≥15 bps were aligned to miRNA sequences in miRBase 21 reference database by bowtie software [25]. The reads aligned to unique location in the reference genome with no more than 2 mismatches were considered as uniquely aligned reads. Raw read counts were normalized as counts per million mappable reads (CPM) using Trimmed Mean of M-values (TMM) method in edgeR software package [26]. Any CPM values <1 were not included in the analysis. Differential expression between groups was analyzed by edgeR using generalized linear model (glm) with empirical Bayes moderation [27].

#### In silico prediction of miRNA target genes

In silico prediction of miRNA targets were performed either in batches or individually. A list of miRNAs in batches was used to predict their putative targets following miRWalk v2.0 [28]. On the other hand, the putative targets of individual miRNAs were predicted using TargetScan v7.2 (http://www.targetscan.org/vert_72/) and miRDB (http://mirdb.org/) databases [29].

#### Determinations of uniquely expressed miRNAs in synaptic fraction and their relative expression ratios (synaptosomal fraction vs. total fraction)

Uniquely expressed miRNAs were defined as those detected in synaptosomes, but not in the total fraction based on the identification criteria discussed in the supplemental section. The expression ratio (synaptosomal/total fraction) of each miRNA was determined independently in control and MDD subjects using normalized CPM values from miRNA-seq data. Subsequently, these values were used to compare synaptosome/total fraction ratios between control and MDD subjects.

#### Functional annotation of miRNA targets following Gene Ontology (GO) prediction

GO analysis was done independently based on predicted targets of miRNAs which were found to be uniquely expressed in synaptosomes as well as those that were significantly dysregulated in synaptic fractions of MDD subjects. To obtain a consensus list of predicted targets, in silico prediction algorithm from 8 different prediction programs (miRWalk, Microt4, miRanda, miRDB, Pictar2, PITA, RNA22, and Targetscan) was used. Next, a common list of target genes, shared by any seven for uniquely expressed and six for significantly dysregulated miRNAs, was used. For miRNAs that were uniquely associated with synaptosomes, the consensus list of predicted target genes was used in standalone Cytoscape program to perform GO analysis using ClueGO plugin. Display pathways were selected with *p* ≤ 0.05. Clustering was done based on common functionality of genes enriched for specific term. The kappa score was set at 0.4 to define the term-term relationship (i.e., edges between the nodes). Genes involved in more than one function were represented with multiple color combination. For synaptosomal miRNAs that were significantly altered in MDD subjects, the consensus list of predicted target genes was used to predict GO terms using the ShinyGO program [30]. The prediction analysis was done following an FDR corrected *p* value cutoff (*p* = 0.05) to determine the gene set enrichment in biological processes (BP) and cellular components (CC) categories separately. In BP, 30 most significant terms were used to plot the network with edge cutoff *p* = 0.05, whereas, in CC, the connected nodes were presented with 40 most significant terms with edge cutoff *p* = 0.03. In each category, the enriched terms were used to create networks where nodes were presented with terms and connected with edges. If two nodes were connected, then they shared 20% (default) or more genes. Bigger nodes represented larger gene sets, while thicker edges represented more overlapped genes. Separately, Ingenuity Pathway Analysis Software (IPA; Qiagen, Valencia, CA, USA) was used to predict the functional role of miRNAs differentially expressed in total fraction of MDD subjects. Modules for functional enrichment of predicted targets deciphering their role in the canonical pathways, molecular networks, and disease pathways were created using Fisher’s exact test and *p* value ≤ 0.05. All visualizations from IPA analysis were made using customized R scripts.

#### qPCR-based miRNA and mRNA-specific gene expression

Primer sequences and qPCR protocols are detailed in the supplemental section (Table S2). miRNA specific cDNA was synthesized using poly(A)-tailing method whereas mRNA-specific cDNA synthesis was performed with the Oligo (dT)_18_ priming method (Invitrogen, USA). qPCR-based relative transcript quantification of all genes was determined with EvaGreen chemistry (Applied Biological Materials, Canada). Pre-miRNA primers were made according to their base sequences (miRBase, http://www.mirbase.org/). Forward primers spanned over the DICER cleavage site and the reverse primer spanned towards 3′ end to make a long product. U6 was used as a normalizer for miRNA transcript quantification, whereas geometric means of GAPDH, ACTB, and ribosomal 18S RNA were applied to quantify synaptosomal mRNAs, pre-miRNAs, and genes associated with miRNA maturation (i.e., DICER1, TARBP2, DROSHA, and AGO2). Fold changes were calculated by Livak’s ΔΔCt method [31].

#### In vitro cell line-based studies

For in vitro experiments, SH-SY5Y neuroblastoma cells (ATCC CRL2266) were used. Double-stranded RNA oligos (miR-19b-3p mimic [C-300483-03-0002]), hairpin inhibitor (IH-300489-05-0002); miR483-5p mimic (C-301107-01-0002), hairpin inhibitor (IH-301107-02-0002), miR-511-5p mimic (C-300752-03-0002), and hairpin inhibitor (IH-300752-05-0002) were purchased (Dharmacon GE Life Sciences, USA) and used. RNA oligos were transfected into SH-SY5Y cells and harvested 48 h post-transfection for target gene expression analysis. The study was replicated in two independent batches of cell lines.

### Statistical analysis

Statistical analyses were conducted using SPSS software (V.25; IBM, USA). Shapiro-Wilk test was used to assess normality of the data. Only data that were normally distributed were used. The differences in age, PMI, and brain pH were assessed by independent-sample *t*-test. Effects of gender, drug abuse, alcohol abuse, and antidepressant medications were analyzed by Fisher’s exact test. The differences in mRNA expression, miRNA expression, and miRNA based relative expression ratios between control and MDD subjects were analyzed using independent-sample *t*-test. The differences in miRNAs and target gene expression among vehicle, mimic, and hairpin inhibitors were assessed by one-way analysis of variance (ANOVA) followed by post-hoc corrections. Correlations of miRNA expression and miRNA ratios of synaptosome/total fraction with covariates were conducted with Pearson correlation coefficient. Statistical significance was set at 95% level (*p* ≤ 0.05).

## Results

### miRNA changes in total and synaptic fractions of MDD subjects

Initially, the synaptic fraction prepared from human dlPFC was characterized. As shown in Fig. S1B, synapsin I was present in total, supernatant, and synaptosomal fractions; PSD-95 was highly enriched in the synaptosomal fraction and was absent in the supernatant fraction. On the other hand, nuclear marker PCNA was absent in the synaptic fraction but was present in the total and supernatant fractions. These results are similar to those reported earlier in mouse [10, 12] and human [32] brain.

**Fig. 1:**
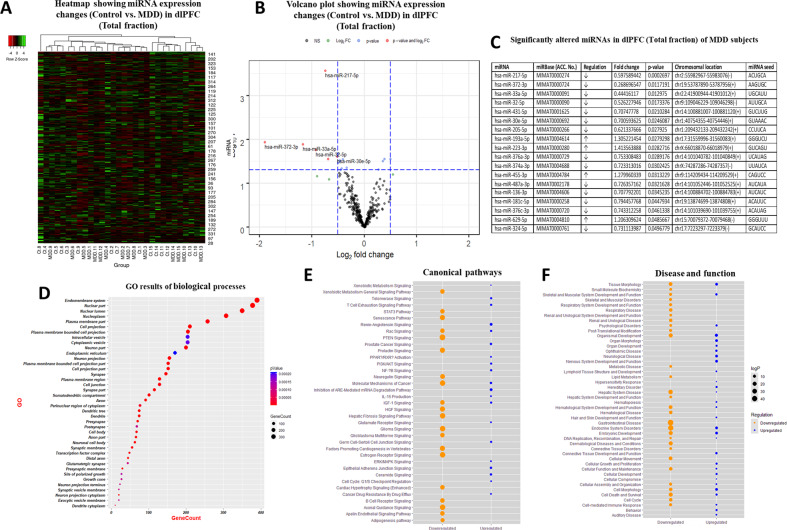
miRNA expression volcano plot, heatmap, gene ontology prediction, and IPA analysis in total fraction. Normalized values of 333 detectable miRNAs were plotted in an expression volcano plot and heatmap with hierarchical clustering. **a** miRNAs with high expression are shown with green color on the map; miRNAs with low expression are shown in red. For the clustering purposes, the Euclidean method was used to measure the distance, and the average linkage algorithm was applied to calculate the average pairwise distance between all pairs of points. Following the average linkage clustering algorithm, the dendrogram was constructed to demonstrate the expression similarities. **b** Volcano plot of genes differentially expressed between control and MDD groups. The *y*-axis corresponds to the significance level represented with log_10_P value, and the *x*-axis displays the log2 (FC) value. The red dots represent the significantly (*p* ≤ 0.05) overexpressed genes in MDD (FC ≥ 1.3); The blue dots represent the significantly (*p* ≤ 0.05) under expressed genes (FC ≤ 1.3) in MDD; the green dots represent the genes whose expression levels did not reach statistical significance (*p* ≥ 0.05) but expression level was higher (FC ≥ 1.3) in MDD group. **c** Significantly altered miRNAs in the total fraction of dlPFC from MDD subjects. Out of 333 miRNAs, 4 miRNAs and 18 miRNAs were significantly up- or downregulated in MDD subjects, respectively. **d** GO analysis for biological process conducted with predicted target genes by significantly altered miRNAs showing significant enrichment of terms in various categories associated with neuronal functions. The lower *p* value is shown as blue color, and the circle size means the number of gene counts in each GO term. IPA analysis was performed with predicted target genes for significantly up- and downregulated miRNAs separately for canonical pathway (**e**) and disease and function (**f**). The results from up- and downregulated miRNA are shown as blue and orange colors respectively.

Both total and synaptic fractions were used for miRNA sequencing separately. In the total fraction, 333 miRNAs were reliably detected following the cleansing of normalized sequencing data. Figure 1a shows an expression heatmap demonstrating the normalized expression levels of select miRNAs following a hierarchical clustering method. Differentially expressed miRNAs in MDD group are shown in Fig. 1b as a volcano plot. Of 333 miRNAs, 4 miRNAs were significantly upregulated and 14 miRNAs were significantly downregulated in MDD subjects compared to control subjects (Fig. 1c).

**Fig. 2:**
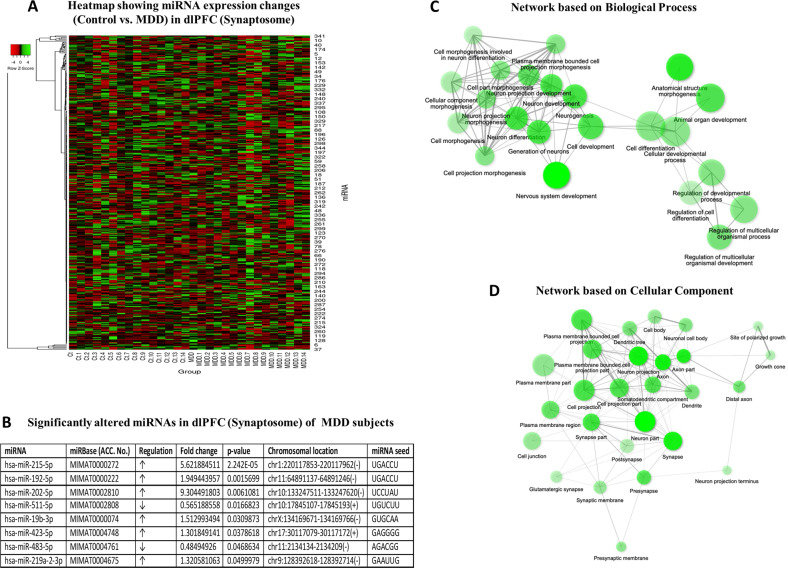
miRNA expression heatmap and GO prediction in the synaptic fraction. **a** Normalized values of 351 miRNAs were plotted in an expression heatmap with hierarchical clustering. miRNAs with high expression are shown with green color on the map whereas miRNAs with low expression are shown in red. For the clustering purpose, the Euclidean method was used to measure the distance, and the average linkage algorithm was applied to calculate the average pairwise distance between all pairs of points. Following the average linkage clustering algorithm, the dendrogram was constructed to demonstrate the expression similarities. **b** Significantly altered miRNAs in the synaptic fraction of dlPFC from MDD subjects. Out of 351 miRNAs, 8 miRNAs were significantly altered in MDD subjects (6 upregulated: miR-215-5p, miR-192-5p, miR-202-5p, miR-19b-3p, miR-423-5p, miR-219a-2-3p; 2 downregulated: miR-511-5p, miR-483-5p). **c** GO analysis for biological process showing significant enrichment of terms in various categories associated with neuronal morphogenesis, growth and differentiation. **d** GO-based functional network using cellular component as predictor, demonstrating significant enrichment of gene sets central to synaptic morphology, function and regulation. The prediction analysis was done following an FDR corrected *p* value cutoff 0.05 to determine the gene set enrichment in biological process and cellular component category separately. In each category the enriched terms were used to create networks where nodes are presented with terms and connected with edges. As shown in the graphs, if two nodes are connected, then they share 20% (default) or more genes. Bigger nodes represent larger gene sets. Thicker edges represent more overlapped genes.

miRNA-seq results in the synaptic fraction showed the expression of 351 miRNAs based on the criteria discussed in the supplemental section. Selected miRNAs expressed in the synaptic fraction are shown in a heat map following a hierarchical clustering method (Fig. 2a). Following the average linkage clustering algorithm, a dendrogram was constructed to demonstrate the expression similarities (Fig. 2a). Of 351 miRNAs expressed in the synaptic fraction, 6 miRNAs (miR-215-5p, miR-192-5p, miR-202-5p, miR-19b-3p, miR-423-5p, miR-219a-2-3p) showed significant upregulation (>30%) and 2 miRNAs (miR-511-5p, miR-483-5p) showed significant downregulation (<50%) in MDD subjects (Fig. 2b).

**Fig. 3:**
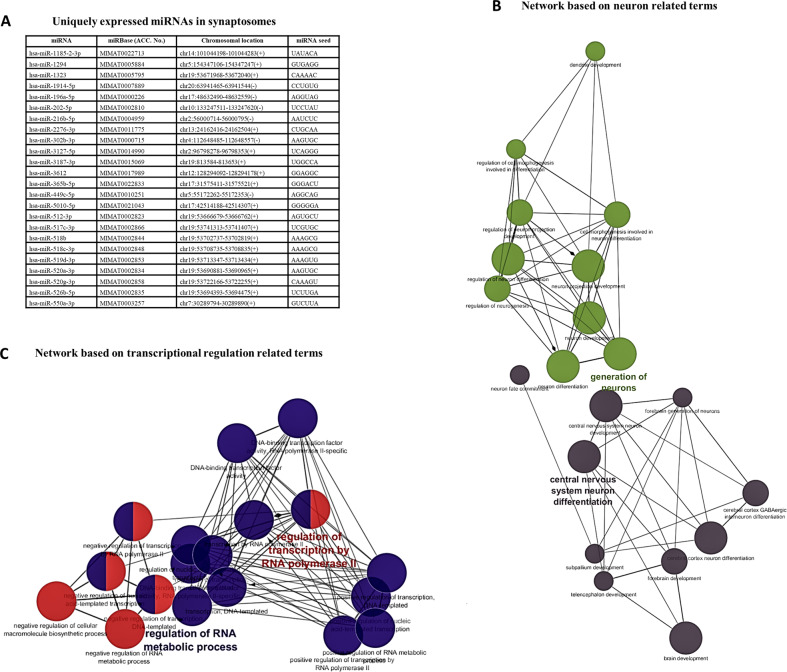
GO prediction based on 24 uniquely expressed miRNAs in synaptosomes. **a** Table shows 24 miRNAs that were uniquely expressed in the synaptic fraction. **b** GO-based functional network using predicted targets of 24 miRNAs recruiting neuro-related terms. Closely connected networks presented with nodes and edges show enrichment of terms related to neuron projection, neuron development, neuron fate commitment and dendritic development. **c** GO-based functional network using predicted targets of 24 miRNAs recruiting transcriptional regulation related terms. Closely connected networks presented with nodes and edges showing enrichment of terms central to transcriptional regulation. A consensus list of predicted targets was used in standalone Cytoscape program to perform the GO analysis. Display pathways were selected with *p* values ≤ 0.05. Clustering was done based on the common functionality of genes enriched for specific term. The kappa score was set at 0.4 to define the term-term relationship (i.e., edges between the nodes). Genes involved in more than one function were represented with multiple color combination.

Next, we determined miRNAs that were uniquely associated with synaptic fraction. It was observed that 24 miRNAs were exclusively expressed in the synaptic fraction and were absent in the total fraction (Fig. 3a). In the pool of uniquely expressed miRNAs, 7 showed >20% upregulation (miR-1294, miR-1914-5p, miR-196a-5p, miR-2276-3p, miR-302b-3p, miR-365b-5p) and 6 showed >20% downregulation (miR-449c-5p, miR-512-3p, miR-517c-3p, miR-519d-3p, miR-520a-3p, miR-550a-3p) in the MDD group; however, only miR-202-5p showed significant change, which was highly upregulated (>9 fold) in the MDD group (Table S3). A phenogram (Fig. S3) was drawn to show the relative localization of uniquely expressed synaptic miRNAs (with 20% change) on different chromosomes. The blue (up) and red (down) colors show miRNAs found with up and downregulated expressions respectively.

### GO and path analysis based on predicted target genes of MDD associated miRNAs in the total fraction

The GO analysis of biological pathways based on in silico predicted target genes of miRNAs from total fraction indicated that they were primarily associated with multiple synapse related GO terms, including axon, dendrites, neuron projection, synaptic vesicle membrane, pre and post-synapse, and glutamatergic synapse (Fig. 1d). The IPA results of canonical and disease pathways are shown as bubble plot (Fig. 1e, f). In canonical pathway, several cellular signaling terms appeared that are highly relevant to depression, such as glutamatergic, ERK/MAPK, neuregulin, estrogen receptor, PI3K, telomeres, as well as axon guidance. The result of disease pathway also indicated that these altered miRNAs were related to nervous systems and psychological disorders (Fig. 1f).

### GO and path analysis based on predicted target genes of miRNAs in the synaptosomal fraction

When GO analysis of 8 significantly altered miRNAs in the synaptosomes of MDD subjects was determined, several important biological pathways and cellular components appeared. As can be seen in Fig. 2c, d, the clustered biological functions were plotted into networks to represent their relatedness. Biological process-based network mapped the term nervous system development as a hub, which was central to other connected terms associated with neurogenesis, neuronal development, differentiation, neuron projection, and morphogenesis. On the other hand, cellular component-based network analysis projected changes in gene functions related to synaptic and somatodendritic compartments. Significant enrichment was also noted for neuronal functions central to morphogenetic changes, including growth cone, dendritic tree, neuron projections, and distal axonic growth. Genes enriched in each category, under biological process and cellular components, are detailed in Tables S4 and S5.

Following GO enrichment analysis several neuronal function-related ontology terms were identified based on predicted targets of 24 uniquely expressed miRNAs from synaptic fraction (Fig. 3a). Top significantly enriched GO terms were associated with neuron projection, neuron development, neuron fate commitment and dendritic development (Fig. 3b). Additionally, network analysis based on the target genes of these miRNAs showed the enrichment of GO terms central to transcriptional regulation (Fig. 3c) which were found to impact RNA metabolism besides influencing neuronal functions.

### In silico prediction and validation of target genes using in vitro cell model

**Fig. 4:**
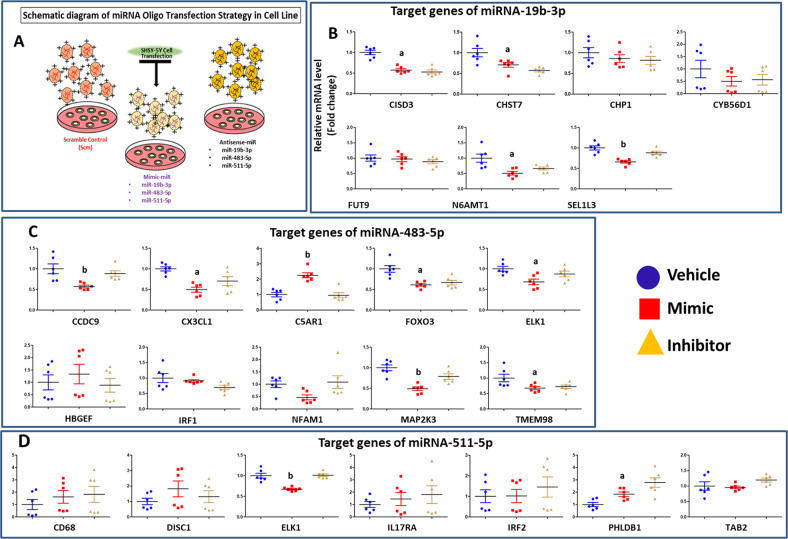
Validation of target genes using in vitro cell model. Relative quantification of target gene expression was done in SH-SY5Y neuroblastoma cell line transiently transfected with vehicle (*n* = 6) or hairpin inhibitors (*n* = 6). **a** Schematic diagram of in vitro study. **b** Mimic miR-19b-3p overexpression oligo (*n* = 6). Overall group differences in the three groups are as follows: CISD: *df* = 2; *f* = 35.2, *p* < 0.01; CHST7: *df* = 2; *f* = 9.9, *p* = 0.002; CHP1: *df* = 2; *f* = 0.8, *p* = 0.451; CYB56D1: *df* = 2; *f* = 1.1, *p* = 0.37; FUT9: *df* = 2; *f* = 0.5, *p* = 0.61; N6AMT1: *df* = 2; *f* = 8.1, *p* = 0.004; SEL1L3: *df* = 2; *f* = 17.8, *p* = 0.001. **c** Mimic miR-483-5p overexpression oligo (*n* = 6). Overall group differences in the three groups are as follows: CCDC9: *df* = 2; *f* = 7.3, *p* = 0.006; CX3CL1: *df* = 2; *f* = 10.8, *p* = 0.001; C5AR1: *df* = 2; *f* = 22.7, *p* < 0.001; FOXO3: *df* = 2; *f* = 12.6, *p* = 0.001; ELK1: *df* = 2; *f* = 23, *p* = 0.001; HBGEF: *df* = 2; *f* = 0.5, *p* = 0.60; IRF1: *df* = 2; *f* = 2.6, *p* = 0.10; NFAM1: *df* = 2; *f* = 3.6, *p* = 0.05; MAP2K3: *df* = 2; *f* = 16.9, *p* < 0.001; TMEM98: *df* = 2; *f* = 4.5, *p* = 0.03. **d** Mimic miR-511-5p overexpression oligo (*n* = 6). Overall group differences in the three groups are as follows: CD68: *df* = 2; *f* = 0.6, *p* = 0.54; DISC1: *df* = 2; *f* = 1.1, *p* = 0.355; ELK1: *df* = 2; *f* = 23, *p* < 0.001; IL17RA: *df* = 2; *f* = 0.6, *p* = 0.57; IRF2: *df* = 2; *f* = 0.4, *p* = 0.65; PHLDB1: *df* = 2; *f* = 8.1, *p* = 0.004; TAB2: *df* = 2; *f* = 17.8, *p* = 0.001. The average differences of target gene expression among vehicle, mimic, and hairpin inhibitor were assessed by one-way ANOVA with post hoc Bonferroni correction. GAPDH was used as normalizer for gene expression. Values denote average ± SEM. ‘a’ and ‘b’ denote statistical significance ‘between vehicle and mimic’ and ‘in mimic compared to vehicle and hairpin inhibitor’, respectively. Inhibitor, hairpin inhibitor.

We randomly selected miRNAs (miR-19b-3p, miR-483-5p, and miR-511-5p) from the list of significantly altered MDD associated miRNAs in synaptosomes (Fig. 2b) and validated their functions in vitro with target gene expression (Fig. 4). Based on TargetScan v7.2 and miRDB databases, several predicted target genes related to neuronal functions were identified. These included: *CISD3, CHP1, CHST7, CYB561D1, FUT9, N6AMT1, SEL1L3* for miR-19b-3p; *C5AR1, CCDC9, CX3CL1, ELK1, FOXO3, HBGEF, IRF1, NFAM1, MAP2K3, TMEM98* for miR-483-5p; and *CD68, DISC1, ELK1, IL17RA, IRF2, PHLDB1, TAB2* for miR-511-5p. The target prediction profile for each miRNA (based on in-silico analysis) is presented in Tables S6, S7, and S8. Significant expression changes of these target genes were confirmed from RNA sequencing determined in the same fraction (Tables S6–8, also see Supplemental section for detailed RNA sequencing methods and analysis). The functional relationship of these genes with corresponding miRNAs (miR-19b-3p, miR-483-5p, and miR-511-5p) was determined by in vitro transfection assay. As shown in Fig. 4, the expression of these genes had an inverse relationship with their corresponding miRNAs. Significant downregulation was found for *CISD3* (43%; *p* < 0.001), *CHST* (29%; *p* = 0.002), *N6AMT1* (49%; *p* = 0.004), and *SEL1L3* (34%; *p* < 0.001) genes in miR-19b-3p mimic transfected cells. Similarly, significant lower expression of *CCDC9* (43%; *p* = 0.006), *CX3CL1* (51%; *p* = 0.001), *ELK1* (32%; *p* = 0.011), *FOXO3* (39%; *p* = 0.001), *MAP2K3* (51%; *p* < 0.001), and *TMEM98* (33%; *p* = 0.030) was noted in miR-483-5p mimic transfected cells. *ELK1* gene was found to be repressed (33%; *p* < 0.001) in miR-511-5p mimic transfected cells. Expression of miR-19b-3p hairpin inhibitor significantly unmasked the repressive effect from *SEL1L3* expression mimic transfected cell. Similar changes were noted for *CCDC9* and *MAP2K3* expressions under the influence of miR-483-5p hairpin inhibitor and *ELK1* expression under the influence of miR-511-5p. Conversely, significant upregulation was found for *C5AR1* (125%; *p* < 0.001) in miR-483-5p mimic and *PHLDB1* (84%; *p* = 0.001) in miR-511-5p mimic transfected cells. However, this trend was not followed by other target genes such as *CHP1*, *CYB56D1*, and *FUT9* for miR-19b-3p mimic; *HBGEF*, *IRF1*, and *NFAM1* for miR-483-5p mimic; and, *CD68*, *DISC1*, *IL17RA* and *IRF2* for miR-511-5p mimic transfected cells.

### Shift in expression ratios of synaptic vs. total miRNAs

A total of 326 miRNAs were commonly expressed in total and synaptosome fractions. Individual miRNAs had a wide range of relative ratios, which was estimated by their expression in control group (Fig. S2). The median synaptic enrichment ratio across all miRNAs was 1.01, with 31.9% of sequences showing enrichment >1.5-fold (19.3% >2-fold) and 32.2% showing depletion >1.5-fold (16.9% ≥2-fold). The highest ratio was found for miR-1908-5p, which was 7.5-fold more abundant in synaptosomes than in total fraction. On the other hand, the lowest ratio was found for miR-101-3p, which was 5.9-fold less abundant in synaptosomes. The top and bottom 20 miRNAs showing high to low ratios in synaptic vs. total fractions are depicted in Table S9.

When the relative expression ratios of miRNAs between synaptic and total fractions were determined, it was observed that the ratios of miR-19b-3p (*p* = 0.047) and miR-376c-3p (*p* = 0.006) were significantly higher, whereas the ratios of miR-455-3p (*p* = 0.012) and miR-337-3p (*p* = 0.038) were significantly lower in MDD subjects compared to control subjects (Table S10). In addition, 20 miRNAs showed large changes in relative expression ratios in MDD subjects (0.36-5.4-fold); however, they could not reach statistical significance (Table S11).

### Expression of miRNA processing enzymes in synaptosomes

qPCR-based expression changes in miRNA processing enzymes were examined in synaptosomes. A trend of increased expression for DROSHA (95%), DICER1 (17%), and TARBP2 (38%) was noted in MDD subjects; however, they were not statistically significant (Fig. S4).

### Pre-miRNA expression changes in synaptosomes

**Fig. 5:**
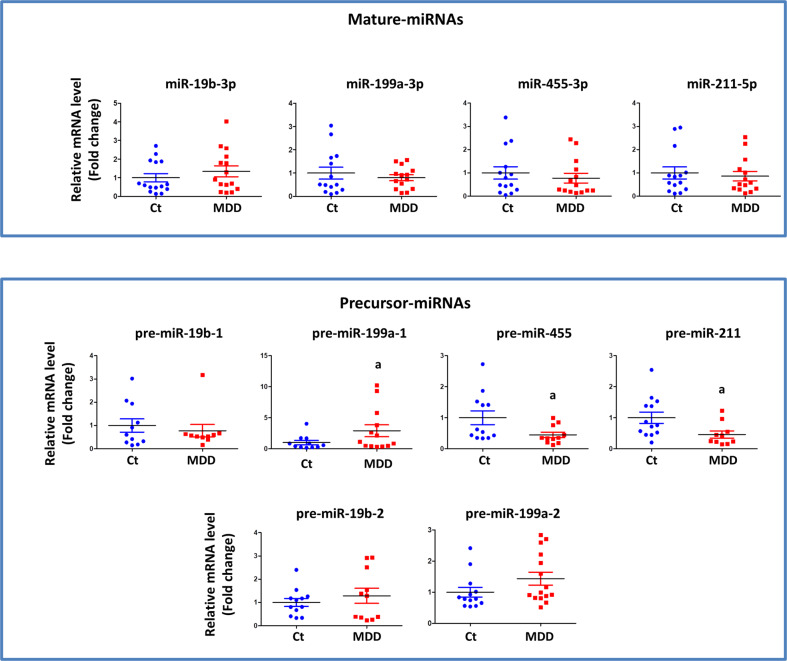
Pre-/mature-miRNA expressions in synaptosomes. Scatter plots represent the relative quantification of mature and their respective precursor miRNAs in synaptosomes. The differences between two groups are as follows: miR-19b-3p: *df* = 8; *t* = −0.949, *p* = 0.35; pre-miR-19b-1: *df* = 19; *t* = 0.57, *p* = 0.57; pre-miR-19b-2: *df* = 21; *t* = −0.79, *p* = 0.43; miR-199a-3p: *df* = 26; *t* = 0.69, *p* = 0.497; pre-miR-199a-1: *df* = 22; *t* = −2.22, *p* = 0.036; pre-miR-199a-2: *df* = 26; *t* = −1.62, *p* = 0.11; miR-455-3p: *df* = 26; *t* = 0.65, *p* = 0.51; pre-miR-455: *df* = 21; *t* = 2.223, *p* = 0.037; miR-215-5p: *df* = 27; *t* = 0.37, *p* = 0.71; pre-miR-215: *df* = 21; *t* = 2.33, *p* = 0.03. The average differences were assessed by student’s *t* test. U6 and geometric means of GAPDH, ACTB, and ribosomal 18S RNA were used as normalizers for mature and precursor miRNAs, respectively. (*n* = 15/group). Ct, control; miRNA, microRNA; MDD, major depressive disorder.

In order to examine if mature miRNAs were derived from their respective pre-miRNAs at synapse, 4 miRNAs (miR-19b-3p, miR-199a-3p, miR-455-3p, miR-211-5p) were randomly selected based on their preferential synaptic expression in MDD subjects as mentioned in Fig. 2b, Table S10, and Table S11. The geometric mean of GAPDH, ACTB, and ribosomal 18S RNA, that was used to normalize the data, was not significantly different between MDD and control groups (*p* = 0.395). Significant changes in the expression of pre-miR-199a-1 (*p* = 0.036), pre-miR-455 (*p* = 0.037), and pre-miR-211 (*p* = 0.030) were noted in MDD subject (Fig. 5). Pre-miR-19b-1, pre-miR-199a-1, and pre-miR-199a-2 had an inverse relationship with their corresponding mature isoforms (miR-19b-3p and miR-199a-3p). On the other hand, miR-455 and miR-211 and their corresponding pre-miRNAs showed changes in the same direction (Fig. 5).

### Effects of confounding variables

Significantly altered miRNAs in total and synaptic fractions were evaluated for their association with confounding variables such as age, sex, brain pH, PMI, antidepressant toxicology, and a history of alcohol and drug abuse. All subjects in control and MDD groups were White except one subject in the control group was Black. Also, only one subject in the MDD group had a history of drug abuse. In the total fraction, there were no significant correlations between brain pH and PMI with any of the altered miRNAs. Age had significant positive correlation with miR19a-3p and significant negative correlations with miR-487-3p, miR-136-3p, and miR-376-3p (Table S11). There were no significant differences in any of the miRNAs between males and females (Table S11). Within the MDD group, 6 subjects had antidepressant positive toxicology. A comparison between those who had positive and negative antidepressant toxicology showed no significant differences in miRNA expression (Table S12). In the MDD group, 3 subjects had a history of alcohol abuse; however, none of the miRNAs showed significant differences when compared with those who did not have a history of alcohol abuse (Table S12).

In the synaptic fraction, age, brain pH and PMI had no significant impact on miRNA expression except for miR-19b-3p which was significantly negatively correlated with age (Table S13). A comparison of males and females showed no significant difference in any of the miRNAs that had altered expression in the MDD group (Table S13). Similarly, antidepressant toxicology did not affect miRNA expression changes in the MDD group (Table S13). A comparison of MDD subjects who had a history of alcohol abuse vs. those who did not also had no significant impact on miRNAs except miR-202-5p which had significantly lower expression in the group showing a history of alcohol abuse (Table S13).

## Discussion

This is the first study to examine the synaptic enrichment of miRNAs and their possible functions in the brain of MDD subjects. We sequenced miRNAs in both total and synaptic fractions obtained from dlPFC of MDD and control subjects to gain insight into miRNA functions at the level of synapse. We chose to examine dlPFC, since numerous brain imaging studies have identified dlPFC as key brain area involved in MDD [33–36]. Frontal cortical activation during inhibitory control also predicts antidepressant treatment response in patients with MDD [37, 38]. In addition, several postmortem brain studies have implicated dlPFC in MDD pathogenesis [9, 39–42]. Our sequencing results in dlPFC showed 333 miRNAs reliably detected in the total fraction of dlPFC; of them, 18 miRNAs were significantly altered in MDD subjects (4 upregulated and 14 downregulated). On the other hand, a total of 351 miRNAs were detectable in the synaptic fraction; out of them, 24 miRNAs were uniquely associated with synaptic fraction. Eight uniquely associated miRNAs were significantly altered in the dlPFC of MDD subjects. In addition, 326 miRNAs were found to be commonly expressed between total and synaptic fractions. Among them, a significant number of miRNAs were either highly enriched or highly depleted in synaptosomes. Also, there were 4 miRNAs that showed a significant shift in their expression ratios between synaptic vs. total fractions in MDD subjects. Additionally, alterations in the expression of pre-miRNAs along with a trend in miRNA maturation and processing enzymes were noted in MDD subjects.

There are broad implications of dysregulated miRNAs in the total and synaptic fractions, alterations in the expression of downstream target genes and their potential functional consequences. When we explored the functions of 18 dysregulated miRNAs in total fraction by GO and IPA analysis with predicted target genes, it was observed that miRNAs that were altered in the total fraction were significantly associated with disruption in a variety of signaling pathways such as PI3K/AKT, ERK/MAPK, Rac, IGF as well as disruption in cell cycle. These findings are quite consistent with earlier reports showing their role in MDD pathogenesis [43–45]. Canonical pathways, molecular networks, and disease pathways also suggested their roles in various neuronal functions and psychological disorders.

We next determined the function of miRNAs that were significantly altered in the synaptic fraction. We found 6 miRNAs (miR-215-5p, miR-192-5p, miR-202-5p, miR-19b-3p, miR-423-5p, miR-219a-2-3p) were significantly upregulated and 2 miRNAs (miR-511-5p, miR-483-5p) were significantly downregulated in MDD subjects. Network-based canonical and biological pathways invariably showed their roles in synaptic plasticity. When individual miRNAs were examined, miR-202-5p and miR-192-5p regulated TGF-β signaling pathway [46–48], which has been implicated in MDD pathophysiology [49]. In the mouse model of depression, miR-192-5p rescues cognitive impairment and restores neural functions by enhancing synaptic transmission and neuronal regeneration via Fbln2/TGF-β1 signaling [47, 50]. Using in vitro system, we examined the regulation of select target genes of miR-19b-3p and miR-511-5p which were significantly altered in MDD subjects. Significant downregulation was found in *CISD3*, *CHST*, *N6AMT1*, and *SEL1L3* genes in miR-19b-3p mimic transfected cells. Among them, N6-methyl-2′-deoxyadenosine methyltransferase (N6AMT1) was associated with the extinction of conditioned fear through the regulation of Bdnf exon IV [51]. On the other hand, *CX3CL1* expression was significantly decreased in miR-483-5p overexpressing cells. miR-483-5p plays a critical role in stress-induced depression, as has been demonstrated in our previous study [52]. *CX3CL1* is highly expressed in neural cells and is necessary for microglial cell migration with the help of CX3CR1 receptor [53, 54]. *CX3CL1* gene induces fractalkine signaling system, which is involved in maturation, activity, and plasticity of developing and mature synapses [53–55] and synaptic repatterning [56]. In fact, CX3CR1-deficient mice show impairment in the maturation of developing glutamatergic synapse in hippocampus [57, 58]. These findings suggest that miR-483-5p and associated fractalkine signaling via *CX3CL1* could be associated with altered synaptic activity reported in MDD subjects [59–61]. miR-511-5p, which was significantly downregulated in MDD subjects, showed an inverse relationship with *ELK1* gene in miR-oligo transfected cell culture model. ELK1 is a transcription factor that is activated upon phosphorylation by ERK. Both ERK and ELK1 have been implicated in MDD [62]. ELK1 integrates pathways of NMDA signaling and glucocorticoid receptor system [63, 64]. It has recently been reported that ELK1 mRNA was upregulated in MDD subjects and the failure to reduce ELK1 expression was associated with resistance to antidepressant treatment [65]. Interestingly, ketamine, a rapid antidepressant, induces spine formation through the activation of mTOR signaling in synaptoneurosomes of rat PFC [66] and treatment with inhibitors of ERK diminishes the behavioral effect of ketamine in forced swim test. In mice, ELK-1 overexpression per se produces depressive behaviors; conversely, the selective inhibition of ELK1 activation prevents depression-like behavior and altered synaptic plasticity induced by stress [65].

As mentioned earlier, we found 24 miRNAs were exclusively present in synaptosomes. Interestingly, 14 miRNAs (8 upregulated and 6 downregulated) showed >20% change in their expression in MDD subjects. GO analysis revealed that these miRNAs were involved in neuronal functions including neuronal projections, neuron development, neuron fate commitment, and dendritic development. Genes (TGFβ, CREBBP, LIMK1, GRIN3A, CACNA1A, NOTCH1, PPP3CA, WNT5A, LEF1, CAMK2, and MAP3K2) that were targets of these miRNAs (Table S14) appeared to be quite relevant in MDD pathophysiology [67–71]. We also mapped these exclusive upregulated and downregulated miRNAs with a schematic phenogram model (Fig. S3) to show their relative position on the respective chromosomes. The phenograpic representation helps to understand if the similarly regulated miRNA loci are closely positioned on a chromosome. We found that downregulated cluster of 4 miRNAs (miR-512-3p, miR-517c-3p, miR-519d-3p and miR-520a-3p) closely shared physical coordinates on chromosome 19. This is important as it has been shown that generally, homologous miRNAs are prone to appear in clusters based on functional and evolutionary relationships. Also, co-expressed miRNAs are mostly members from a single polycistronic transcript and share common target genes and participate in a particular biological pathway and disease pathophysiology [72]. In fact, we have shown earlier that a large number of miRNAs were downregulated in rat prefrontal cortex which showed resiliency to develop depression, and all showed a blunted response to those that had a susceptibility to depression phenotype. All miRNAs were encoded at a few shared polycistronic loci suggesting that their downregulation was coordinately controlled at the level of transcription. Interestingly, most of these miRNAs have previously been shown to be enriched in synaptic fractions [10, 11]. In the future, it will be interesting to examine the chromosomal organization vis-à-vis genomic clustering of synaptic miRNAs and their functional correlates.

Our study also showed not only a high enrichment of a large number of miRNAs in synaptosomes, but also a shift in miRNAs in synaptic fraction of MDD subjects when ratios of total vs. synaptic miRNAs were determined. It has previously been shown that miRNAs are not only highly enriched near synapse and expressed within dendrites in mammalian brain, but are also regulated in an activity-dependent manner thereby participating in plasticity responses [73]. In fact, pri-miRNAs are present in synaptic fractions and are especially enriched in isolated post-synaptic densities [11]. DROSHA and DGCR8 proteins are also expressed at synapse and are tightly associated with pri-miRNAs [11]. pri-miRNAs are also transported to synaptic regions and their processing occurs locally near synapses in a regulated fashion [11]. We found a shift in the expression of several miRNAs (e.g., miR-376c-3p, miR-455-3p and miR-337-3p) in the synaptic fraction over total fraction in MDD subjects compared with healthy controls. In addition, miRNAs (e.g., miR-19b-3p and miR-199a-3p) had an inverse relationship with their corresponding pre-miRNAs in the synapse. These findings suggest that miRNA biogenesis/maturation may be occurring at synapse and the availability of miRNA biogenesis machinery may be disrupted in MDD. Interestingly, we found a trend in the alterations in miRNA processing enzymes DROSHA, DICER1, and TARBP2 in MDD subjects. It has been reported that DICER is located in dendrites and axons [11–13, 74] and pre-miRNAs are cleaved into mature miRNAs near synapses [13, 15, 74]. In addition, we also found that miR-455-3p and miR-211-5p and their corresponding pre-miRNAs were both downregulated. This is quite interesting as it has been reported earlier that biogenesis enzymes need several co-factors to be activated [75]. In addition, specific conditions are necessary to transport and process individual synaptosomal pre-miRNAs. For example, in addition to DROSHA, the conversion of pre-miR-134 to mature miR-134 in dendrites needs DEAH-box helicase DHX36 binding to its terminal loop [13]. We speculate that the co-factors related to biogenesis enzyme activation and/or transportation of certain pre-miRNAs to synaptosome may not be functional in MDD as was noted for miR-455-3p and miR-211-5p where both pre-miRNA and mature miRNAs were downregulated. Alternatively, we hypothesize that the shift in expression could be due to dysregulated distribution and partitioning of expressed miRNAs between total and synaptic compartments under MDD pathology. Further studies will be needed to confirm these speculations.

In conclusion, our study, for the first time, shows that a large number of miRNAs are synaptically enriched and a pool of miRNAs are uniquely associated with synapse. These synaptic miRNAs are differentially regulated in MDD subjects. In addition, there is a shift in the expression of synaptically enriched miRNAs, suggesting that miRNAs may be processed locally at synapse and this processing may be aberrant in MDD brain. Altogether, our findings add a new dimension to understanding MDD pathogenesis. As suggested earlier, the shift in miRNAs may be due to altered expression and/or functions of miRNA biogenesis machinery at synapse. The enzymes and co-factors that are involved in miRNA biogenesis may possibly serve as potential therapeutic targets for future drug development in the treatment of MDD.

## Funding and disclosures

This work was supported by the National Institutes of Health (R01MH082802; R01MH101890; R01MH100616; R01MH107183; R01MH118884) to YD. The authors report no other financial interests or potential conflicts of interest.

## Supplementary information

Supplemental materials
