# Supplementary material for: Differential and unique patterns of synaptic miRNA expression in dorsolateral prefrontal cortex of depressed subjects
Source: Neuropsychopharmacology. 2020 Sep 12;46(5):900–10. doi: 10.1038/s41386-020-00861-y (PMC8115313; doi:10.1038/s41386-020-00861-y)
Supplement: Supplementary file 1 — Supplemental materials [file 41386_2020_861_MOESM1_ESM.docx]

**Supplemental Section**

**Differential and unique patterns of synaptic miRNA expression in dorsolateral prefrontal cortex of depressed subjects**

Yuta Yoshino, M.D., Ph.D., Bhaskar Roy, Ph.D., Yogesh Dwivedi, Ph.D.

Department of Psychiatry and Behavioral Neurobiology

University of Alabama at Birmingham, Birmingham, Alabama, 35294, USA

**Running Title:** Synaptic miRNAs and depression pathophysiology

***Corresponding author:**

Yogesh Dwivedi, Ph.D.

Elesabeth Ridgely Shook Professor

Director of Translational Research, UAB Mood Disorder Program

Co-Director, UAB Depression and Suicide Center

Department of Psychiatry and Behavioral Neurobiology

University of Alabama at Birmingham

SC711 Sparks Center

1720 7^th^ Avenue South

Birmingham, Alabama, USA

Phone: 01-205-975-8459

Email: [yogeshdwivedi@uabmc.edu](mailto:ydwivedi@uabmc.edu)

***Human postmortem brain studies***

***Subjects***

The study was approved by the Institutional Review Board of the University of Alabama at Birmingham. Brain tissues were obtained from the Alabama Brain Collection program as well as Maryland Brain Collection program, Baltimore. Family members/informants signed written informed consents. Psychiatric diagnoses of the subjects were made after receiving written informed consent from at least one member/informant from the family, who underwent an interview based on the Diagnostic Evaluation After Death (DEAD) [[1](#_ENREF_1)] and the Structured Clinical Interview for the DSM-IV (SCID) [[2](#_ENREF_2),[3](#_ENREF_3)]. The interviews were done by a trained psychiatric social worker. Two psychiatrists independently reviewed the write-up from this interview, as well as the SCID that was completed from it, as part of their diagnostic assessment of the case. Diagnoses were made from the data obtained in this interview, medical records from the case, and records obtained from the Medical Examiner's office. The two diagnoses were compared, and discrepancies were resolved by means of a consensus conference. Control subjects were verified as free from mental illnesses using these consensus diagnostic procedures by psychological autopsy using DSM-V criteria by means of SCID-I interviews [[2](#_ENREF_2)]. SCID-I is a structured diagnostic procedure to elicit diagnostic information by means of proxy-based interviews complemented with medical and coroner records, followed by a consensus diagnosis reached by a panel of clinicians using DSM-V criteria. Both cases and controls were characterized by the same psychological autopsy methods, therefore avoiding the occurrence of systematic biases. The demographic and clinical characteristics of subjects are provided in **Table S1**. Demographic data include age, gender, race, postmortem interval (PMI), brain pH, cause of death, race, past history of drug abuse, alcohol abuse, and antidepressant medication. Family members/informants signed written informed consent.

***Postmortem brain***

The study was performed in dorsolateral prefrontal cortex (dlPFC; Brodmann area [BA] 46) obtained from right hemisphere of MDD and control subjects (n = 15/group). After removal from the cranium, the brains were cut into six major pieces (four cerebral cortical lobes, basal ganglia-diencephalon, and lower brain stem-cerebellum), rapidly frozen on dry ice, and stored at -70°C until dissection. During dissection, the frontal lobes were sliced into 1mm to 1.5 mm thick coronal sections at a temperature between 0°C to 10°C. To keep the samples frozen, the dissections were performed on a metal plate over a container filled with dry ice. The prefrontal cortical samples were cut out of the coronal sections by a fine microdissecting (Graefe) knife under a stereomicroscope with low magnification. BA46 was taken just dorsal to the frontopolar area including the most polar portion of the superior and partly the middle frontal gyrus between the superior and intermediate frontal sulci. In the sections of the dissected cortical area, the gray and white matters were separated. The tissues were chopped into smaller pieces after flash-frozen in isopentene at -80°C and later stored at -80°C until use. All tissues from control subjects and MDD subjects were screened for evidence of neuropathology by experienced neuropathologists. The presence of Alzheimer disease, infarcts, demyelinating diseases, or atrophy disqualified subjects from the study. Toxicology data were obtained by the analysis of urine and blood samples.

***Synaptosome preparation***

Synaptosome fraction was prepared following the methods of Smallheiser and Collins [[4](#_ENREF_4)] and Lugli et al. [[5](#_ENREF_5)] with slight modifications. Briefly, 100mg of tissues were homogenized manually by Fisherbrand™ RNase-Free Disposable Pellet Pestles (Fisher Scientific, USA) in homogenizing buffer (HB) (50mM HEPES, pH 7.5, 125mM NaCl, 100mM Sucrose, 10mM EDTA, pH 8.0, 2mM PMSF, 1x Halt Protease Inhibitor Cocktail, 160U/ml RNaseOUT). The lysate was collected as a total fraction. Later supernatant (S fraction) was collected after centrifugation at 20,000g for 20 min at 4’C. The pellet was resuspended with HB buffer and used in a sucrose gradient centrifugation step to obtain synaptosome fraction with 0.32, 0.85, 1.0, and 1.2M sucrose in 1mM NaHCO3 and 160U/ml RNaseOUT. Synaptosome fraction was recovered in 1.0-1.2M interface after centrifugation at 200,000g for 2 hr at 4^0^C in Optima XPN-100 Ultracentrifuge (Beckman Coulter, Indianapolis, IN, USA) SW 41 rotor and Ultra-Clear Centrifuge Tubes (Beckman Coulter, Indianapolis, IN, USA). Purified synaptosome fraction was aliquoted in two parts and stored in -80’C temperature a) as synaptosome lysate for protein assays and b) lysate mixed with TRIzol ® (Invitrogen, Grand Island, NY, USA) for RNA isolation.

***Protein expression analysis following Western blot method***

Protein lysate was prepared by methanol/chloroform precipitation and dissolve in RIPA buffer (Tris-Cl [pH 8.0] 50mM, NaCl 150mM, IGEPAL (NP-40) 1%, Sodium Deoxycholate 0.5%, SDS 0.1%, EDTA 2.5mM supplemented with 1X complete protease inhibitor, 1mM of PMSF [phenylmethylsulfonyl fluoride] and 25μm of MG-132). Those samples were subject to immunoblot analysis after resolving on denatured discontinuous SDS–polyacrylamide gel electrophoresis (SDS-PAGE). Probing with primary antibody was performed after validating the ideal concentration of primary antibodies for detection of specific protein on blot [(PCNA (1:2000, Cell Signaling #2586), PSD95 (1:1000, Cell Signaling #2507), and Synapsin I (1:3000, Cell Signaling #5297)]. Horseradish peroxidase (HRP)-conjugated secondary antibody (Applied Biological Materials Inc., Canada) was used separately for each primary antibody to develop blot images. All the antibodies were diluted in 2% non-fat milk in TBST (1L of 1x tris-buffered saline and 1 mL of Tween 20 [polysorbate 20]). For capturing the images, the membranes were exposed chemiluminescent signal using Dark box Image Capture and Analysis System (Syngene-G: Box, USA).

***Isolation of RNA from total and synaptosome fractions***

TRIzol ® (Invitrogen, Grand Island, NY, USA) was used to isolate RNA as described earlier [[6](#_ENREF_6)]. RNA purity was checked by Nanodrop (260/280 nm; cutoff ≥ 1.8) and their integrity by agarose gel electrophoresis. The RIN for samples were >7.

***Library construction and sequencing of total RNA using synaptosomal RNA sample***

Transcriptome-wide expression of total RNA in synaptosome was measured using next generation sequencing platform from Arraystar, Inc. (MD, USA). RNA sample quantity and quality were initially tested on a NanoDrop ND-1000 instrument (ThermoScientific, Waltham, MA, USA). Agarose electrophoresis was used to check the integrality of total RNA samples. 1-2ug total RNA from each sample was taken for RNA-seq library preparation. Briefly, mRNA was isolated from total RNA with NEBNext® Poly(A) mRNA Magnetic Isolation Module (New England Biolabs, Ipswich, MA, USA). Alternatively, rRNA was removed from the total RNA with a RiboZero Magnetic Gold Kit (epicenter, Madison, WI, USA); The enriched mRNA or rRNA depleted RNA was used for RNA-seq library preparation using KAPA Stranded RNA-Seq Library Prep Kit (Illumina, Indianapolis, IN, USA). The library preparation procedure included: 1) fragmentation of the RNA molecules; 2) reverse transcription to synthesis first strand cDNA; 3) second strand cDNA synthesis incorporating dUTP; 4) end-repair and A-tailing of the double stranded cDNA; 5) Illumina compatible adapter ligation; and 6) PCR amplification and purification for the final RNA-seq library. The completed libraries were qualified on Agilent 2100 Bioanalyzer for concentration, fragment size distribution between 400-600bp, and adapter dimer contamination. The amount was determined by absolute quantification qPCR method. The barcoded libraries were mixed in equal amounts and used for sequencing on the instrument. The DNA fragments in well mixed libraries were denatured with 0.1M NaOH to generate single-stranded DNA molecules, loaded onto channels of the flow cell at 8pM concentration, and amplified in-situ using TruSeq SR Cluster Kit v3-cBot-HS (#GD-401-3001, Illumina, USA). Sequencing was carried out using the Illumina HiSeq 4000 (Illumina, USAs) according to the manufacturer’s instructions. Sequencing was carried out by running 150 cycles.

***Bio-computational analysis of RNA sequencing data***

Raw data files in FASTQ format were generated from the Illumina sequencer. To examine the sequencing quality, the quality score plot of each sample was plotted. Sequence quality was examined using the FastQC software. After quality control, the fragments were 5’, 3’-adaptor trimmed and filtered ≤ 20 bp reads with cutadapt software. The trimmed reads were aligned to reference genome with Hisat 2 software. Based on alignment statistical analysis (mapping ratio, rRNA/mtRNA content, fragment sequence bias), we determine whether the results can be used for subsequent data analysis. The expression level (FPKM value) of known genes and transcripts were calculated using ballgown through the transcript abundances estimated with StringTie. The number of identified genes and transcripts per group was calculated based on the mean of FPKM in group ≥ 0.5. Differentially expressed gene and transcript analyses were performed with R package ballgown.

***Library construction and sequencing of miRNAs***

miRNA based transcriptomic expression in synaptosome was measured using next generation sequencing platform from Arraystar, Inc. (MD, USA). Total RNA prepared from purified synaptosome was used to prepare the miRNA sequencing library for each sample. Briefly, the NGS library was prepared using NEB Multiplex Small RNA Library Prep Set for Illumina (New England Biolabs, Ipswich, MA, USA), which included the following steps: (1) 3'-adapter ligation by T4 RNA ligase 2; (2) 5'-adapter ligation by T4 RNA ligase; (3) cDNA synthesis by reverse transcription; (4) low cycle PCR amplification of the library DNA; (5) Size selection by polyacrylamide gel electrophoresis of 135~155bp PCR amplified fragments (corresponding to ~15-35nt small RNAs). After the libraries were prepared for each sample, they were quantified with Agilent 2100 Bioanalyzer to determine the quality of the library. Next, the DNA fragments in the libraries were denatured with alkaline treatment (0.1M NaOH) to generate single-stranded DNA molecules, captured on Illumina flow cells, amplified in-situ, and finally sequenced for 51 cycles on Illumina NextSeq 500 (Illumina, USA) according to the manufacturer’s instruction. Raw sequencing data generated from Illumina NextSeq 500 that pass the Illumina chastity filter are used for following analysis.

***Bio-computational analysis of miRNA sequencing data***

The raw sequencing reads were removed of the adapter sequence as the trimmed reads by cutadapt software. Reads ≥ 15 bps were aligned to the miRNA sequences in the miRBase 21 reference database by bowtie software [[7](#_ENREF_7)]. The reads aligned to unique location in the reference genome with no more than 2 mismatches were considered as uniquely aligned reads. Raw read counts were normalized as counts per million mappable reads (CPM) using Trimmed Mean of M-values (TMM) method in edgeR software package [[8](#_ENREF_8)]. Differential expression between groups was analyzed by edgeR using generalized linear model (glm) with empirical Bayes moderation [[9](#_ENREF_9)].

***Total RNA isolation from human dlPFC and transcript level expression analysis following qPCR method***

The extracted RNAs were reverse transcribed into first-strand cDNA using M-MLV Reverse Transcriptase (Invitrogen, Grand Island, NY, USA). A mixture of 0.5µg of total RNA, oligo (dT)_18_, dNTP, and double distilled water was incubated at 65°C for 5 minutes and quickly chilled on the ice. Subsequently, the reaction mixture was added with 1 x 1st strand synthesis buffer, 0.01mM DTT, 2U of RNaseOut and 200U of M-MLV reverse transcriptase (Invitrogen, Grand Island, NY, USA) and incubated at 37°C for 50 min. Finally, the reaction was inactivated at 70°C. qPCR was carried out in 96-well plates with an Mx3005P qPCR machine (Stratagen, La Jolla, CA, USA) using 1x EvaGreen qPCR MasterMix (Applied Biological Material, Richmond, BC, Canada). Each reaction mixture contained 5μl of 40-fold diluted cDNA, 10μl of EvaGreen 2x qPCR MasterMix, 0.8μM of each primer, and nuclease-free water to a final volume of 25μl. The thermal cycling program was shown in **Supplemental Table S2,** which was conducted with 96-well optical reaction plates (USA Scientific, Ocala, FL, USA). The melting curves were analyzed at 59-95°C after 40 cycles. The amplification products were checked on 3% agarose gels. Each qRT-PCR analysis was performed with two technical replicates for miRNA biogenesis study and three technical replicates for cell culture study. Relative gene expression level was quantified after normalizing the Ct values with respective normalizers (geometric means [GAPDH, ACTB, and ribosomal 18S] for miRNA biogenesis study and GAPDH for cell culture study) and fold change values were determined following 2^- ΔΔCt^ calculation method [[10](#_ENREF_10)]. Some samples were excluded from qPCR results because those values were significant outliers according to a web-based outlier calculator (https://www.miniwebtool.com/outlier-calculator/).

***Cell culture and in vitro transfection experiments with miRNA oligo***

SH-SY5Y cells were cultured in DMEM containing 10% fetal bovine serum, 2mM glutamine, and penicillin and streptomycin (10000U/ml). The cells were incubated at 37°C in a 5% CO2 atmosphere and the medium were refreshed every 24h. Transient transfections of miRNAs mimics or hairpin inhibitor (Dharmacon, Lafayette, CO, USA) were performed using Lipofectamine RNAiMAX (Invitrogen, Grandsland, NY, USA) according to the manufacturer’s protocol.

***Target gene prediction for validation assay using in vitro system***

Target gene prediction by miRNA was conducted by TargetScan v7.2 (http://www.targetscan.org/vert_72/) and miRDB (http://www.mirdb.org/). First, we listed all of target genes, which were hit by at least one database. Second, we narrowed down those target genes based on the following criteria: target genes that have inverse relationship between the expression of target genes determined by RNA-seq and the expression of miRNA (miR-19b-3p, miR-483-5p, and miR-511-5p, respectively) determined by miRNA-seq (**Table S7-9**). Finally, we selected 7-10 genes related to brain function for cell culture studies.

**Table S1. Demographic and clinical characteristics of control and MDD subjects**

|  | | **Control** | **MDD** | **p value** |
| --- | --- | --- | --- | --- |
| Number of subjects | | 15 | 15 |  |
| Age (Years) | | 57.0 ± 15.0 | 57.9 ± 14.0 | 0.871 |
| Gender | Male | 8 | 7 | N/A |
|  | Female | 7 | 8 |  |
| Postmortem interval (Hour) | | 18.1 ± 6.0 | 17.8 ± 6.8 | 0.877 |
| Brain pH | | 6.3 ± 0.8 | 6.5 ± 0.5 | 0.408 |
| Race (white: black: other) | | 14: 1: 0 | 15: 0: 0 | N/A |
| Drug abuse | | 0 | 1 | N/A |
| Alcohol abuse | | 0 | 3 | N/A |
| Antidepressant drugs | | 0 | 6 (Amitriptyline, paroxetine, alprazolam, quetiapine, quetiapine quetiapine, doxepine, bupropion, trazodone) | N/A |
| Suicide | | 0 | 0 | N/A |
| Cause of death | | Pulmonary embolism, lymphoma, cardiac failure, lung cancer, acute myocardial infarction, cardiopulmonary arrest, cerebrovascular accident, colon cancer, leukemia, renal failure, cardiac arrest | Cardiopulmonary arrest, pneumonia, cerebrovascular accident, cardiac failure, cardiac arrest, leukemia, heart disease, multiple vehicle accident, liver failure | N/A |

Values denote mean ± standard deviation. MDD: major depressive disorder, N/A: not applicable.

| **Table S2. Prime sequences and thermal cycling parameters for qPCR assays** | | | | |  |
| --- | --- | --- | --- | --- | --- |
| **Primer name** | **Primer Orientation** | **Sequence** | **cDNA dilution** | **Primer concentration (µM)** | **Thermal parameters** |
| **miRNA cDNA synthesis** |  |  |  |  |  |
| Oligo dT Adapter Primer V3 |  | GCGAGCACAGAATTAATACGACTCACTATAGGTTTTTTTTTTTTTTTTTTVN |  |  |  |
|  |  |  |  |  |  |
| **miRNA qPCR** |  |  |  |  |  |
| hsa_miR-19b-3p | F | TGCAAATCCATGCAAAACTGAA | 30x | 0.5 | (95’C for 10sec, 55’C for 15 sec, 20 sec for 20 sec) x 40 cycle, 95’C for 1 min, 55’C for 30 sec, 95’C for 30 sec |
| hsa_miR-483-5p | F | AAGACGGGAGGAAAGAAG | 30x | 0.5 |  |
| hsa_miR-511-5p | F | GTCTTTTGCTCTGCAGTCAAA | 30x | 0.5 |  |
| hsa_miR-199a-3p | F | ACAGTAGTCTGCACATTGGTTA | 30x | 0.8 |  |
| hsa_miR-215-5p | F | ATGACCTATGAATTGACAGACAAAAAA A | 30x | 0.5 |  |
| hsa_miR-423-5p | F | GCAGAGAGCGAGACTTTAAAAAAA | 30x | 0.5 |  |
| hsa_miR-219-3p | F | ATG TGG CTG GAC ATC TGT AA | 30x | 0.5 |  |
| has_miR-455-3p | F | AGTCCATGGGCATATACACAA | 30x | 0.5 | (95’C for 10sec, 58’C for 15 sec, 20 sec for 20 sec) x 40 cycle, 95’C for 1 min, 55’C for 30 sec, 95’C for 30 sec |
| hsa-miR-211-5p | F | CTTTGTCATCCTTCGCCTAAAAA | 30x | 0.5 | (95’C for 10sec, 58’C for 20 sec, 20 sec for 20 sec) x 40 cycle, 95’C for 1 min, 55’C for 30 sec, 95’C for 30 sec |
| hsa-miR-192-5p | F | GAC CTATGAATTGACAGCCAAAAA | 30x | 0.5 |  |
| hsa-miR-202-5p | F | TTCCTATGCATATACTTCTTTGAAAAAAAAAA | 30x | 0.5 |  |
| Universal Reverse |  | GCGAGCACAGAATTAATACGAC |  | same as F primer |  |
| U6 | F | CTCGCTTCGGCAGCACA | 30x | 0.8 | (95’C for 10sec, 55’C for 15 sec, 20 sec for 20 sec) x 40 cycle, 95’C for 1 min, 55’C for 30 sec, 95’C for 30 sec |
|  | R | AACGCTTCACGAATTTGCGT |  |  |  |
|  |  |  |  |  | |
| **Pre-miRNA qPCR** |  |  |  |  |  |
| hsa-pre-miR-19b-1 | F | GTT TGCATC CAGCTGTGT | 20x | 0.8 | 95’C for 10 sec, (95’C for 10sec, 55’C for 15 sec, 20 sec for 20 sec) x 40 cycle, 95’C for 1 min, 55’C for 30 sec, 95’C for 30 sec |
|  | R | CACTACCACAGT CAGTTTTGC |  |  |  |
| hsa-pre-miR-19b-2 | F | CAGGTTTGCATTTCAGCGT |  |  |  |
|  | R | CATTATCACAATCAGTTTTGCATGG |  |  |  |
| hsa-pre-miR-199a-1 | F | GTTCAGACTACCTGTTCAGGA |  |  |  |
|  | R | CCTAACCAATGTGCAGACTACT |  |  |  |
| hsa-pre-miR-199a-2 | F | CCAGTGTTCAGACTACCT |  |  |  |
|  | R | TCTCCCTTGCCCAGTC |  |  |  |
| hsa-pre-miR-455 | F | CCTTTGGACTACATCGTGGA |  |  |  |
|  | R | ATGACATAGGCCTTGAGGC |  |  |  |
| hsa-pre-miR-211 | F | TTGTCATCCTTCGCCTAGG |  |  |  |
|  | R | TGCTGTGGGAAGTGACAA |  |  |  |
| hsa-miR-106a | F | TTACAGTGCAGGTAGCTTTTTG |  |  |  |
|  | R | GTAAGAAGTGCTTACATTGCAGT |  |  |  |
|  |  |  |  |  |  |
| **mRNA qPCR** |  |  |  |  |  |
| DROSHA | F | CCCAGATTGTGACCCTAGACTT | 20x | 0.8 | 95’C for 10 sec, (95’C for 10sec, 60’C for 15 sec, 20 sec for 20 sec) x 40 cycle, 95’C for 1 min, 55’C for 30 sec, 95’C for 30 sec |
|  | R | ATGAACAGCCACCGGATGAAA |  |  |  |
| DICER1 | F | CCCCACTTTAGAGCCCTGTG |  |  |  |
|  | R | GCCTCCCCAGTCCTTTACAC |  |  |  |
| TARBP2 | F | AGATCATGGCAGGCAGCAAG |  |  |  |
|  | R | TACATGAGCCCAGAGGCAGA |  |  |  |
| AGO2 | F | ACTCTGTTCCGCCCTTTAGC |  |  |  |
|  | R | CAGGCACCAAGAGACCATGT |  |  |  |
| CISD3 | F | TCCCACTTCTTCCAACGCAC |  |  |  |
|  | R | GAGTGGCCTTGCAGGTACAG |  |  |  |
| CHP1 | F | ACATCTGCCCTTGGTTGACT |  |  |  |
|  | R | CCTGGCTACTCCCCTGTTCT |  |  |  |
| CHST7 | F | TAGCTCCGTCTCCCTCTACC |  |  |  |
|  | R | ATTAGGGTTTTCCCAGCCCAG |  |  |  |
| CYB56D1 | F | ATGCTCCTGTGTCTGGTTGG |  |  |  |
|  | R | ACACCAGTGATAGGGACGGA |  |  |  |
| FUT9 | F | GCATCCTGGGAGCAAAGCAT |  |  |  |
|  | R | TGGTTGGTAAAGTCGGCGTC |  |  |  |
| N6AMT1 | F | AAGGTGACAGGCACAAGCCTA |  |  |  |
|  | R | AATGACCTAGGCAAGTCAGAGGC |  |  |  |
| SEL1L3 | F | AGCCCTGTTCCTCAAAGCTG |  |  |  |
|  | R | ATTGCCCTCCCTGCATTTCC |  |  |  |
| C5AR1 | F | ACCATCTTTCCATCCCAGGC |  |  |  |
|  | R | TCCCTGTTCACACCTATTGCC |  |  |  |
| CCDC9 | F | ATGCCCGTCTCTGGAATGTC |  |  |  |
|  | R | ATTCTTTATTGGGGAGGGGGC |  |  |  |
| CX3CL1 | F | GTTCTCTGCCCTCCAAGACC |  |  |  |
|  | R | CTTCCCCTTTCCCATGTCCC |  |  |  |
| ELK1 | F | TGGAGTTGGGAGGGAGGAAT |  |  |  |
|  | R | AAATCACCAACCCCCAGGTC |  |  |  |
| FOXO3 | F | CAGAAATACGTGTGCGGCTG |  |  |  |
|  | R | CAAGAGAAGACTGATTGCCAGG |  |  |  |
| HBGEF | F | AATCTGGCTTAGTGCCACCC |  |  |  |
|  | R | GCACTCTGACCACGGAAGAT |  |  |  |
| IRF1 | F | CACCAGGGCTGTCAGTTGAT |  |  |  |
|  | R | TGGAGCAGCATCTGTGTGTT |  |  |  |
| MAP2K3 | F | CAGGGGCTATGGGTTTGCTT |  |  |  |
|  | R | CCCCAACCCATCAGGGAAAG |  |  |  |
| TMEM98 | F | TAGTTGTTCTCCACGGCTGG |  |  |  |
|  | R | GAGGCATAAACTGACGGGGA |  |  |  |
| CD68 | F | ATGAGATAATGCAGTCGGGAG |  |  |  |
|  | R | GGACAAGGAAAACCCCGTCA |  |  |  |
| DISC1 | F | GAAACTGAGCCTGGGGCAAA |  |  |  |
|  | R | CCTGGGATGACTGGGGAATG |  |  |  |
| IL17RA | F | GGTCACAACACAGAGAGGGG |  |  |  |
|  | R | ACTGGGCCTGCACCAAAATA |  |  |  |
| IRF2 | F | GAGTGGATGCCTCAGAACGG |  |  |  |
|  | R | ACTGCAATGTCGCTAGTGCT |  |  |  |
| PHLDB1 | F | CATCATCTCTCCAGCCCAGG |  |  |  |
|  | R | AGGCAGGGGGAAGGAAAAAG |  |  |  |
| TAB2 | F | GCCCTAACTTTCAGGCTTTGC |  |  |  |
|  | R | TCAAACGTAAGGGCTTTGGGT |  |  |  |
| GAPDH | F | CCACATCGCTGAGACACCAT |  |  |  |
|  | R | AGTTAAAAGCAGCCCTGGTGA |  |  |  |
| ACTB | F | AGTCATTCCAAATATGAGATGCGTT |  |  |  |
|  | R | TGCTATCACCTCCCCTGTGT |  |  |  |
| ribosomal 18S RNA | F | CGGCGACGACCCATTCGAAC |  |  |  |
|  | R | GAATCGAACCCTGATTCCCCGTC |  |  |  |
| Primer orientation: Forward primer (F); Reverse primer (R) | | | | | |

|  | **Table S3. miRNAs showing > 20% change that are uniquely expressed in synaptosomes** | | | | | | | |
| --- | --- | --- | --- | --- | --- | --- | --- | --- |
| **miRNA** | | **miRBase (ACC. No.)** | **Regulation** | **Fold change** | **p value** | **Chromosomal location** | **miRNA seed** |  |
| **Upregulation** | |  |  |  |  |  |  |  |
| hsa-miR-1294 | | MIMAT0005884 | ↑ | 1.276104394 | 0.39666721 | chr5:154347106-154347247(+) | GUGAGG |  |
| hsa-miR-1914-5p | | MIMAT0007889 | ↑ | 1.279651524 | 0.266234002 | chr20:63941465-63941544(-) | CCUGUG |  |
| hsa-miR-196a-5p | | MIMAT0000226 | ↑ | 1.579876688 | 0.20233854 | chr17:48632490-48632559(-) | AGGUAG |  |
| hsa-miR-202-5p | | MIMAT0002810 | ↑ | 9.304491803 | 0.006108061 | chr10:133247511-133247620(-) | UCCUAU |  |
| hsa-miR-2276-3p | | MIMAT0011775 | ↑ | 1.260571505 | 0.348150887 | chr13:24162416-24162504(+) | CUGCAA |  |
| hsa-miR-302b-3p | | MIMAT0000715 | ↑ | 1.33256112 | 0.264093722 | chr4:112648485-112648557(-) | AAGUGC |  |
| hsa-miR-3187-3p | | MIMAT0015069 | ↑ | 1.252637532 | 0.27561892 | chr19:813584-813653(+) | UGGCCA |  |
| hsa-miR-365b-5p | | MIMAT0022833 | ↑ | 1.26864889 | 0.176502147 | chr17:31575411-31575521(+) | GGGACU |  |
|  | |  |  |  |  |  |  |  |
| **Downregulation** | |  |  |  |  |  |  |  |
| hsa-miR-449c-5p | | MIMAT0010251 | ↓ | 0.78902145 | 0.279650929 | chr5:55172262-55172353(-) | AGGCAG |  |
| hsa-miR-512-3p | | MIMAT0002823 | ↓ | 0.677533191 | 0.105904059 | chr19:53666679-53666762(+) | AGUGCU |  |
| hsa-miR-517c-3p | | MIMAT0002866 | ↓ | 0.682276568 | 0.256001434 | chr19:53741313-53741407(+) | UCGUGC |  |
| hsa-miR-519d-3p | | MIMAT0002853 | ↓ | 0.767554649 | 0.423648284 | chr19:53713347-53713434(+) | AAAGUG |  |
| hsa-miR-520a-3p | | MIMAT0002834 | ↓ | 0.739657586 | 0.247511206 | chr19:53690881-53690965(+) | AAGUGC |  |
| hsa-miR-550a-3p | | MIMAT0003257 | ↓ | 0.716420991 | 0.272803184 | chr7:30289794-30289890(+) | GUCUUA |  |

**Table S4. Ontology (Biological Process) based gene set enrichment of 8 altered miRNAs in synaptosomal fraction**

| **Functional category** | **Genes in list** | **Total genes** | **Enrichment FDR** |
| --- | --- | --- | --- |
| Nervous system development | 289 | 2474 | 1.4E-28 |
| Anatomical structure morphogenesis | 299 | 2785 | 1.3E-23 |
| Neuron differentiation | 182 | 1412 | 1.8E-21 |
| Neurogenesis | 204 | 1683 | 2.6E-21 |
| Generation of neurons | 195 | 1575 | 2.6E-21 |
| Neuron development | 157 | 1154 | 8.8E-21 |
| Cell development | 246 | 2230 | 1.1E-20 |
| Animal organ development | 361 | 3779 | 1.2E-20 |
| Neuron projection development | 140 | 1008 | 2.9E-19 |
| Positive regulation of metabolic process | 355 | 3789 | 8.0E-19 |
| Positive regulation of cellular metabolic process | 331 | 3482 | 3.0E-18 |
| Regulation of multicellular organismal development | 231 | 2138 | 3.1E-18 |
| Positive regulation of macromolecule metabolic process | 331 | 3498 | 5.4E-18 |
| Neuron projection morphogenesis | 104 | 666 | 1.1E-17 |
| Regulation of developmental process | 276 | 2763 | 1.3E-17 |
| Regulation of multicellular organismal process | 321 | 3382 | 1.3E-17 |
| Plasma membrane bounded cell projection morphogenesis | 105 | 680 | 1.3E-17 |
| Cell differentiation | 396 | 4459 | 1.3E-17 |
| Cell part morphogenesis | 107 | 701 | 1.3E-17 |
| Cell projection morphogenesis | 105 | 682 | 1.5E-17 |
| Cellular developmental process | 409 | 4671 | 2.8E-17 |
| Positive regulation of nitrogen compound metabolic process | 317 | 3351 | 3.1E-17 |
| Tube development | 138 | 1062 | 9.7E-17 |
| Tube morphogenesis | 119 | 860 | 2.4E-16 |
| Cell-cell signaling | 195 | 1774 | 4.7E-16 |
| Cell morphogenesis | 136 | 1067 | 8.3E-16 |
| Cellular component morphogenesis | 145 | 1172 | 8.3E-16 |
| Response to organic substance | 325 | 3547 | 9.6E-16 |
| Cell morphogenesis involved in neuron differentiation | 92 | 598 | 2.5E-15 |
| Regulation of cell differentiation | 206 | 1954 | 3.9E-15 |

**Table S5. Ontology (Cellular Components) based gene set enrichment of 8 altered miRNAs in synaptosomal fraction**

| **Functional category** | **Genes in list** | **Total genes** | **Enrichment FDR** |
| --- | --- | --- | --- |
| Neuron part | 200 | 1808 | 2.2E-16 |
| Axon | 92 | 639 | 2.0E-13 |
| Neuron projection | 155 | 1371 | 2.0E-13 |
| Synapse | 146 | 1268 | 2.5E-13 |
| Axon part | 66 | 392 | 9.2E-13 |
| Synapse part | 115 | 1004 | 3.1E-10 |
| Plasma membrane bounded cell projection | 205 | 2214 | 1.1E-09 |
| Cell projection | 210 | 2287 | 1.1E-09 |
| Presynapse | 70 | 512 | 2.3E-09 |
| Cell projection part | 152 | 1523 | 2.4E-09 |
| Plasma membrane bounded cell projection part | 152 | 1523 | 2.4E-09 |
| Endomembrane system | 390 | 4988 | 2.9E-09 |
| Somatodendritic compartment | 101 | 879 | 2.9E-09 |
| Plasma membrane region | 129 | 1278 | 3.2E-08 |
| Nucleoplasm | 307 | 3861 | 1.2E-07 |
| Distal axon | 45 | 293 | 1.2E-07 |
| Nuclear part | 378 | 4966 | 1.6E-07 |
| Nuclear lumen | 350 | 4545 | 2.1E-07 |
| Dendrite | 75 | 647 | 4.2E-07 |
| Dendritic tree | 75 | 649 | 4.5E-07 |
| Neuronal cell body | 63 | 517 | 9.0E-07 |
| Neuron projection cytoplasm | 20 | 85 | 2.1E-06 |
| Presynaptic membrane | 29 | 165 | 2.8E-06 |
| Plasma membrane part | 257 | 3250 | 3.7E-06 |
| Cell body | 67 | 594 | 5.4E-06 |
| Transcription factor complex | 49 | 385 | 6.7E-06 |
| Cell junction | 129 | 1414 | 6.7E-06 |
| Synaptic membrane | 55 | 459 | 8.8E-06 |
| Perinuclear region of cytoplasm | 77 | 746 | 2.1E-05 |
| Dendrite cytoplasm | 11 | 34 | 4.8E-05 |
| Glutamatergic synapse | 44 | 366 | 1.0E-04 |
| Growth cone | 27 | 178 | 1.1E-04 |
| Synaptic vesicle membrane | 20 | 110 | 1.1E-04 |
| Exocytic vesicle membrane | 20 | 110 | 1.1E-04 |
| Neuron projection terminus | 23 | 139 | 1.1E-04 |
| Postsynapse | 68 | 667 | 1.1E-04 |
| Site of polarized growth | 27 | 181 | 1.3E-04 |
| Cytoplasmic vesicle | 204 | 2625 | 1.9E-04 |
| Intracellular vesicle | 204 | 2628 | 2.0E-04 |
| Endoplasmic reticulum | 170 | 2123 | 2.1E-04 |

**Table S6. *In silico* predicted targets of miR-19b-3p**

|  |  | **RNA-seq results** | | **TargetScan** | | | **miRDB** |
| --- | --- | --- | --- | --- | --- | --- | --- |
| **Target gene** | **Gene name** | **Fold change** | **p value** | **Cumulative weighted context++ score** | **Total context++ score** | **Aggregate PCT** | **Target score** |
| SEL1L3 | SEL1L Family Member 3 | 0.844103 | 0.00246 | -0.06 | -0.16 | 0.83 | 78 |
| CISD3 | CDGSH iron sulfur domain 3 | 0.783335 | 0.008777 | -0.01 | -0.37 | 0.45 | 50 |
| N6AMT1 | N-6 adenine-specific DNA methyltransferase 1 | 0.794196 | 0.019206 |  |  |  | 84 |
| CHST7 | Carbohydrate Sulfotransferase 7 | 0.744365 | 0.022626 | -0.01 | -0.23 | 0.58 |  |
| CYB561D1 | cytochrome b561 family member D1 | 0.74271 | 0.025408 | -0.37 | -0.39 | 0.85 | 61 |
| FUT9 | fucosyltransferase 9 | 0.792413 | 0.026134 | 0.25 | -0.25 | 0.83 | 94 |
| LZIC | Leucine Zipper And CTNNBIP1 Domain Containing | 0.878577 | 0.035074 | -0.20 | -0.28 | 0.76 |  |
| SGCD | Sarcoglycan Delta | 0.844713 | 0.035106 | -0.15 | -0.19 | 0.58 |  |
| TRPM3 | Transient Receptor Potential Cation Channel Subfamily M Member 3 | 0.815773 | 0.035236 | -0.03 | -0.07 | < 0.1 |  |
| CHP1 | calcineurin like EF-hand protein 1 | 0.731093 | 0.038248 |  |  |  | 80 |
| WNT7B | Wnt family member 7B | 0.777049 | 0.039821 | -0.25 | -0.25 | 0.83 | 55 |
| ASAP2 | ArfGAP with SH3 domain, ankyrin repeat and PH domain 2 | 0.879024 | 0.042663 | -0.08 | -0.25 | 0.60 | 88 |
| PDE7B | phosphodiesterase 7B | 0.887171 | 0.049076 | -0.22 | -0.22 | 0.78 | 59 |

RNA-Seq column showing the overlapping presence of matched target genes determined from total RNA sequencing in dlPFC (synaptic fraction) of MDD subjects.

**Table S7. *In silico* predicted targets of miR-483-5p**

|  |  | **RNA-seq results** | | **TargetScan** | | |
| --- | --- | --- | --- | --- | --- | --- |
| **Target gene** | **Gene name** | **Fold change** | **p value** | **Cumulative weighted context++ score** | **Total context++ score** | **Aggregate PCT** |
| AC006486.1 |  | 1.506194 | 0.005838 | -0.31 | -0.31 | N/A |
| RPS6KA2 | Ribosomal Protein S6 Kinase A2 | 1.210914 | 0.010976 | -0.02 | -0.02 | N/A |
| MAP2K3 | Mitogen-Activated Protein Kinase Kinase 3 | 1.4013 | 0.012284 | -0.24 | -0.24 | N/A |
| CX3CL1 | C-X3-C Motif Chemokine Ligand 1 | 1.650968 | 0.01643 | -0.15 | -0.16 | N/A |
| HBEGF | Heparin Binding EGF Like Growth Factor | 1.407104 | 0.017895 | 0.23 | -0.23 | N/A |
| TOR4A | Torsin Family 4 Member A | 1.24805 | 0.019227 | -0.06 | -0.06 | N/A |
| EIF3F | Eukaryotic Translation Initiation Factor 3 Subunit F | 1.210193 | 0.026459 | -0.09 | -0.35 | N/A |
| FAM114A1 | Family With Sequence Similarity 114 Member A1 | 1.22047 | 0.027164 | 0 | -0.14 | N/A |
| GDE1 | Glycerophosphodiester Phosphodiesterase 1 | 1.216708 | 0.027864 | -0.02 | -0.21 | N/A |
| C5AR1 | Complement C5a Receptor 1 | 1.34836 | 0.029767 | -0.23 | -0.23 | N/A |
| NFAM1 | NFAT Activating Protein With ITAM Motif 1 | 1.288658 | 0.035989 | -0.17 | -0.17 | N/A |
| NRBP2 | Nuclear Receptor Binding Protein 2 | 1.183885 | 0.036836 | -0.11 | -0.11 | N/A |
| KLHL36 | Kelch Like Family Member 36 | 1.099693 | 0.038656 | 0 | -0.22 | N/A |
| CCDC9 | Coiled-Coil Domain Containing 9 | 1.115372 | 0.04155 | -0.27 | -0.29 | N/A |
| LEMD2 | LEM Domain Containing 2 | 1.264203 | 0.045934 | -0.13 | -0.13 | N/A |
| KRBA2 | KRAB-A Domain Containing 2 | 1.145246 | 0.046001 | -0.15 | -0.23 | N/A |
| FOXO3 | Forkhead Box O3 | 1.209633 | 0.04748 | -0.14 | -0.14 | N/A |
| TMEM98 | Transmembrane Protein 98 | 1.276224 | 0.048118 | -0.34 | -0.34 | N/A |
| RALY | RALY Heterogeneous Nuclear Ribonucleoprotein | 1.127119 | 0.048589 | -0.04 | -0.35 | N/A |
| AC018755.1 |  | 1.19383 | 0.048827 | -0.06 | -0.28 | N/A |
| ELK1 | ETS Transcription Factor ELK1 | 1.279285 | 0.049417 | -0.02 | -0.02 | N/A |
| IRF1 | Interferon Regulatory Factor 1 | 1.289512 | 0.049636 | -0.01 | -0.22 | N/A |

**Table S8. *In silico* predicted targets of miR-511-5p**

|  |  | **RNA-seq results** | | **TargetScan** | | | **miRDB** |
| --- | --- | --- | --- | --- | --- | --- | --- |
| **Target gene** | **Gene name** | **Fold change** | **p value** | **Cumulative weighted context++ score** | **Total context++ score** | **Aggregate PCT** | **Target score** |
| ECHDC3 | Enoyl-CoA Hydratase Domain Containing 3 | 1.431142 | 0.00083 | 0 | -0.04 | N/A |  |
| ZBED3 | Zinc Finger BED-Type Containing 3 | 1.345427 | 0.0009 | -0.02 | -0.03 | N/A |  |
| IRF2 | Interferon Regulatory Factor 2 | 1.303134 | 0.005163 | -0.10 | -0.10 | N/A |  |
| SMOC1 | SPARC Related Modular Calcium Binding 1 | 1.536705 | 0.006264 | 0 | -0.09 | N/A |  |
| BHLHE41 | Basic Helix-Loop-Helix Family Member E41 | 1.271709 | 0.008505 | -0.03 | -0.08 | N/A |  |
| EMILIN2 | Elastin Microfibril Interfacer 2 | 1.225245 | 0.009287 | -0.02 | -0.02 | N/A |  |
| DISC1 | DISC1 Scaffold Protein | 1.185792 | 0.011087 | -0.01 | -0.01 | N/A | 93 |
| SLCO1A2 | Solute Carrier Organic Anion Transporter Family Member 1A2 | 1.327741 | 0.011124 | -0.03 | -0.03 | N/A |  |
| DUSP16 | Dual Specificity Phosphatase 16 | 1.273927 | 0.011259 | -0.02 | -0.02 | N/A |  |
| JPT2 | Jupiter Microtubule Associated Homolog 2 | 1.288628 | 0.011967 |  |  |  | 57 |
| RASSF2 | Ras Association Domain Family Member 2 | 1.338023 | 0.012546 | -0.03 | -0.03 | N/A |  |
| SOX8 | SRY-Box 8 | 1.324671 | 0.012971 | -0.03 | -0.03 | N/A |  |
| GTPBP1 | GTP Binding Protein 1 | 1.175353 | 0.013574 | -0.02 | -0.02 | N/A |  |
| SMAD9 | SMAD Family Member 9 | 1.28823 | 0.014751 | -0.05 | -0.05 | N/A |  |
| AKIRIN1 | Akirin 1 | 1.394551 | 0.017537 | 0 | -0.05 | N/A |  |
| H2AFJ | H2A Histone Family Member J | 1.274047 | 0.020564 | 0 | -0.02 | N/A |  |
| RGS16 | Regulator Of G Protein Signaling 16 | 1.686076 | 0.020914 | -0.02 | -0.02 | N/A |  |
| PHLDB1 | Pleckstrin Homology Like Domain Family B Member 1 | 1.411454 | 0.022394 | -0.04 | -0.05 | N/A | 65 |
| IL17RA | Interleukin 17 Receptor A | 1.248752 | 0.022523 | 0 | -0.02 | N/A |  |
| ZNF570 | Zinc Finger Protein 570 | 1.217345 | 0.02264 | 0 | -0.03 | N/A |  |
| TRIM62 | Tripartite Motif Containing 62 | 1.231059 | 0.023785 | -0.02 | -0.02 | N/A |  |
| CSF3R | Colony Stimulating Factor 3 Receptor | 1.237778 | 0.023827 | -0.02 | -0.20 | N/A |  |
| PTPRC | Protein Tyrosine Phosphatase Receptor Type C | 1.29264 | 0.025011 | 0 | -0.02 | N/A |  |
| EIF3F | Eukaryotic Translation Initiation Factor 3 Subunit F | 1.210193 | 0.026459 | -0.01 | -0.02 | N/A |  |
| GALNT15 | Polypeptide N-Acetylgalactosaminyltransferase 15 | 1.627819 | 0.02682 | -0.02 | -0.04 | N/A |  |
| N4BP2L1 | NEDD4 Binding Protein 2 Like 1 | 1.321333 | 0.02695 | -0.01 | -0.01 | N/A |  |
| SPATA13 | Spermatogenesis Associated 13 | 1.31132 | 0.027528 | -0.02 | -0.02 | N/A |  |
| FNBP1 | Formin Binding Protein 1 | 1.183708 | 0.028259 | 0 | -0.02 | N/A | 61 |
| BMP8B | Bone Morphogenetic Protein 8b | 1.216091 | 0.02943 | -0.02 | -0.02 | N/A |  |
| TAB2 | TGF-Beta Activated Kinase 1 (MAP3K7) Binding Protein 2 | 1.234626 | 0.030371 | -0.10 | -0.23 | N/A |  |
| TCF12 | Transcription Factor 12 | 1.224607 | 0.030526 | -0.02 | -0.03 | N/A |  |
| MTSS1 | MTSS I-BAR Domain Containing 1 | 1.228194 | 0.03125 | -0.01 | -0.01 | N/A |  |
| CD59 | CD59 Molecule (CD59 Blood Group) | 1.389239 | 0.031705 | 0 | -0.04 | N/A |  |
| CDK19 | Cyclin Dependent Kinase 19 | 1.142423 | 0.031834 | -0.03 | -0.04 | N/A | 79 |
| SIGLEC9 | Sialic Acid Binding Ig Like Lectin 9 | 1.376795 | 0.032149 |  |  |  | 58 |
| WASF2 | WASP Family Member 2 | 1.308276 | 0.032357 | -0.02 | -0.02 | N/A |  |
| RCAN1 | Regulator Of Calcineurin 1 | 1.15949 | 0.033967 | 0 | -0.04 | N/A |  |
| OTUD7B | OTU Deubiquitinase 7B | 1.31744 | 0.037051 | -0.03 | -0.03 | N/A |  |
| STAT5A | Signal Transducer And Activator Of Transcription 5A | 1.180101 | 0.037164 | -0.03 | -0.03 | N/A |  |
| TIFA | TRAF Interacting Protein With Forkhead Associated Domain | 1.178567 | 0.037615 | -0.12 | -0.21 | N/A |  |
| TNFRSF10B | TNF Receptor Superfamily Member 10b | 1.307972 | 0.039262 | -0.01 | -0.01 | N/A |  |
| PLA2G16 | Phospholipase A And Acyltransferase 3 | 1.25269 | 0.039581 | 0 | -0.32 | N/A |  |
| SHROOM4 | Shroom Family Member 4 | 1.380761 | 0.040091 | -0.04 | -0.11 | N/A |  |
| SNIP1 | Smad Nuclear Interacting Protein 1 | 1.158711 | 0.040705 | -0.01 | -0.02 | N/A |  |
| SHMT1 | Serine Hydroxymethyltransferase 1 | 1.267793 | 0.041061 | 0 | -0.01 | N/A |  |
| CD68 | CD68 Molecule | 1.57713 | 0.042399 | -0.23 | -0.23 | N/A |  |
| RNASET2 | Ribonuclease T2 | 1.346231 | 0.044216 | 0 | -0.01 | N/A |  |
| CDKN1C | Cyclin Dependent Kinase Inhibitor 1C | 1.336935 | 0.044649 | -0.02 | -0.02 | N/A | 59 |
| SH3PXD2B | SH3 And PX Domains 2B | 1.445258 | 0.045021 | -0.06 | -0.06 | N/A | 66 |
| CHD2 | Chromodomain Helicase DNA Binding Protein 2 | 1.227499 | 0.045567 | -0.02 | -0.02 | N/A |  |
| KRBA2 | KRAB-A Domain Containing 2 | 1.145246 | 0.046001 | -0.17 | -0.18 | N/A | 80 |
| BAZ2A | Bromodomain Adjacent To Zinc Finger Domain 2A | 1.144142 | 0.048275 | -0.02 | -0.06 | N/A | 66 |
| BMF | Bcl2 Modifying Factor | 1.197748 | 0.048578 | -0.08 | -0.10 | N/A |  |
| RALY | ALY Heterogeneous Nuclear Ribonucleoprotein | 1.127119 | 0.048589 | 0 | -0.01 | N/A |  |
| TMEM187 | Transmembrane Protein 187 | 1.188729 | 0.048711 | -0.16 | -0.16 | N/A |  |
| ELK1 | ETS Transcription Factor ELK1 | 1.279285 | 0.049417 | -0.01 | -0.02 | N/A |  |

**Table S9. Synaptosome/total fraction ratio of miRNA expressions in control subjects (top and bottom 20)**

| **miRNA** | **miRBase (ACC. No.)** | **Synaptosome/total fraction ratio** | **Chromosomal location** | **miRNA seed** |
| --- | --- | --- | --- | --- |
| **Top 20** |  |  |  |  |
| hsa-miR-1908-5p | MIMAT0007881 | 7.5491 | chr11:61815161-61815240(-) | GGCGGG |
| hsa-miR-517a-3p | MIMAT0002852 | 6.8180 | chr19:53712268-53712354(+) | UCGUGC |
| hsa-miR-517b-3p | MIMAT0002857 | 6.8180 | chr19:53721076-53721142(+) | UCGUGC |
| hsa-miR-505-5p | MIMAT0004776 | 5.0950 | chrX:139924148-139924231(-) | GGAGCC |
| hsa-miR-885-5p | MIMAT0004947 | 4.9265 | chr3:10394489-10394562(-) | CCAUUA |
| hsa-miR-3180-3p | MIMAT0015058 | 4.8152 | chr16:14911220-14911313(+) | GGGGCG |
| hsa-miR-92b-3p | MIMAT0003218 | 4.7958 | chr1:155195177-155195272(+) | AUUGCA |
| hsa-miR-766-5p | MIMAT0022714 | 4.7207 | chrX:119646738-119646848(-) | GGAGGA |
| hsa-miR-328-3p | MIMAT0000752 | 4.7052 | chr16:67202321-67202395(-) | UGGCCC |
| hsa-miR-3622a-5p | MIMAT0018003 | 4.2009 | chr8:27701677-27701759(+) | AGGCAC |
| hsa-miR-487a-5p | MIMAT0026559 | 4.0567 | chr14:101052446-101052525(+) | UGGUUA |
| hsa-miR-3173-5p | MIMAT0019214 | 3.9211 | chr14:95137919-95137986(-) | GCCCUG |
| hsa-miR-1343-3p | MIMAT0019776 | 3.7111 | chr11:34941837-34941920(+) | UCCUGG |
| hsa-miR-346 | MIMAT0000773 | 3.6036 | chr10:86264694-86264788(-) | GUCUGC |
| hsa-miR-485-5p | MIMAT0002175 | 3.5114 | chr14:101055419-101055491(+) | GAGGCU |
| hsa-miR-744-5p | MIMAT0004945 | 3.5109 | chr17:12081899-12081996(+) | GCGGGG |
| hsa-miR-433-3p | MIMAT0001627 | 3.4977 | chr14:100881886-100881978(+) | UCAUGA |
| hsa-miR-935 | MIMAT0004978 | 3.4648 | chr19:53982307-53982397(+) | CAGUUA |
| hsa-miR-193b-5p | MIMAT0004767 | 3.4326 | chr16:14303967-14304049(+) | GGGGUU |
| hsa-miR-671-3p | MIMAT0004819 | 3.4154 | chr7:151238421-151238538(+) | CCGGUU |
|  |  |  |  |  |
| **Bottom 20** |  |  |  |  |
| hsa-miR-106b-5p | MIMAT0000680 | 0.3166 | chr7:100093993-100094074(-) | AAAGUG |
| hsa-miR-590-3p | MIMAT0004801 | 0.3121 | chr7:74191198-74191294(+) | AAUUUU |
| hsa-miR-136-3p | MIMAT0004606 | 0.3117 | chr14:100884702-100884783(+) | AUCAUC |
| hsa-miR-19a-3p | MIMAT0000073 | 0.3108 | chr13:91350891-91350972(+) | GUGCAA |
| hsa-miR-451a | MIMAT0001631 | 0.3042 | chr17:28861369-28861440(-) | AACCGU |
| hsa-miR-340-5p | MIMAT0004692 | 0.2958 | chr5:180015303-180015397(-) | UAUAAA |
| hsa-miR-539-3p | MIMAT0022705 | 0.2950 | chr14:101047321-101047398(+) | UCAUAC |
| hsa-miR-127-3p | MIMAT0000446 | 0.2657 | chr14:100882979-100883075(+) | CGGAUC |
| hsa-miR-20b-5p | MIMAT0001413 | 0.2653 | chrX:134169809-134169877(-) | AAAGUG |
| hsa-miR-376c-3p | MIMAT0000720 | 0.2629 | chr14:101039690-101039755(+) | ACAUAG |
| hsa-miR-106a-5p | MIMAT0000103 | 0.2532 | chrX:134170198-134170278(-) | AAAGUG |
| hsa-miR-542-3p | MIMAT0003389 | 0.2523 | chrX:134541341-134541437(-) | GUGACA |
| hsa-miR-651-5p | MIMAT0003321 | 0.2396 | chrX:8126965-8127061(+) | UUAGGA |
| hsa-miR-19b-3p | MIMAT0000074 | 0.2300 | chr13:91351192-91351278(+) | GUGCAA |
| hsa-miR-153-3p | MIMAT0000439 | 0.2241 | chr7:157574336-157574422(-) | UGCAUA |
| hsa-miR-143-3p | MIMAT0000435 | 0.2231 | chr5:149428918-149429023(+) | GAGAUG |
| hsa-miR-374a-3p | MIMAT0004688 | 0.2199 | chrX:74287286-74287357(-) | UUAUCA |
| hsa-miR-32-5p | MIMAT0000090 | 0.2090 | chr9:109046229-109046298(-) | AUUGCA |
| hsa-miR-34c-5p | MIMAT0000686 | 0.2018 | chr11:111513439-111513515(+) | GGCAGU |
| hsa-miR-101-3p | MIMAT0000099 | 0.1688 | chr1:65058434-65058508(-) | ACAGUA |

| **Table S10. Significantly altered ratios of synaptosome/total fraction miRNAs in MDD subjects** |
| --- |

| **miRNAs** | **miRBase (ACC. No.)** | **Synaptosome/total fraction Ratio** | | | **p value** | **Chromosomal location** |
| --- | --- | --- | --- | --- | --- | --- |
|  |  | **Control subjects** | **MDD subjects** | **MDD/Control** |  |  |
| hsa-miR-19b-3p | MIMAT0000074 | 0.249 ± 0.127 | 0.472 ± 0.394 | 1.863421 | 0.047 | chr13:91351192-91351278(+) |
| hsa-miR-376c-3p | MIMAT0000720 | 0.308 ± 0.174 | 0.505 ± 0.191 | 1.745031 | 0.006 | chr14:101039690-101039755(+) |
| hsa-miR-455-3p | MIMAT0004784 | 1.366 ± 0.315 | 1.077 ± 0.270 | 0.759194 | 0.012 | chr9:114209434-114209529(+) |
| hsa-miR-337-3p | MIMAT0000754 | 1.836 ± 0.337 | 1.470 ± 0.559 | 0.775263 | 0.038 | chr14:100874493-100874585(+) |

**Table S11. Comparison of synaptosome/total fraction miRNA ratio between control and MDD subjects (based on fold change)**

| **miRNA** | **miRBase (ACC. No.)** | **Chromosomal location** | **Ct ratio** | **MDD ratio** | **MDD/Ct** | **p value** |
| --- | --- | --- | --- | --- | --- | --- |
| **High ratio in MDD** |  |  |  |  |  |  |
| hsa-miR-215-5p | MIMAT0000272 | chr1:220117853-220117962(-) | 0.515017 | 2.812396 | 5.460783 | 0.18 |
| hsa-miR-372-3p | MIMAT0000724 | chr19:53787890-53787956(+) | 1.400222 | 3.486638 | 2.49006 | 0.143 |
| hsa-miR-192-5p | MIMAT0000222 | chr11:64891137-64891246(-) | 0.550453 | 1.254829 | 2.279628 | 0.184 |
| hsa-miR-200a-3p | MIMAT0000682 | chr1:1167863-1167952(+) | 1.024855 | 2.080298 | 2.029847 | 0.157 |
| hsa-miR-429 | MIMAT0001536 | chr1:1169005-1169087(+) | 0.779552 | 1.559422 | 2.000408 | 0.208 |
| hsa-miR-144-3p | MIMAT0000436 | chr17:28861533-28861618(-) | 0.746424 | 1.459662 | 1.955539 | 0.119 |
| hsa-miR-19b-3p | MIMAT0000074 | chrX:134169671-134169766(-) | 0.249329 | 0.47194 | 1.892836 | 0.047 |
| hsa-miR-376c-3p | MIMAT0000720 | chr14:101039690-101039755(+) | 0.30808 | 0.505447 | 1.640637 | 0.006 |
| hsa-miR-217-5p | MIMAT0000274 | chr2:55982967-55983076(-) | 0.550911 | 0.841181 | 1.526891 | 0.088 |
|  |  |  |  |  |  |  |
| **Low ratio in MDD** |  |  |  |  |  |  |
| hsa-miR-214-3p | MIMAT0000271 | chr1:172138798-172138907(-) | 1.061795 | 0.707184 | 0.666027 | 0.394 |
| hsa-miR-199a-3p | MIMAT0000232 | chr19:10817426-10817496(-) | 1.05151 | 0.695674 | 0.661595 | 0.121 |
| hsa-miR-199b-3p | MIMAT0004563 | chr9:128244721-128244830(-) | 1.051123 | 0.69498 | 0.661179 | 0.121 |
| hsa-miR-199a-3p | MIMAT0000232 | chr19:10817426-10817496(-) | 1.051123 | 0.694936 | 0.661136 | 0.121 |
| hsa-miR-516b-5p | MIMAT0002859 | chr19:53725442-53725526(+) | 4.374716 | 2.672417 | 0.610878 | 0.345 |
| hsa-miR-2116-3p | MIMAT0011161 | chr15:59171183-59171262(-) | 3.010654 | 1.812771 | 0.602119 | 0.309 |
| hsa-miR-211-5p | MIMAT0000268 | chr15:31065032-31065141(-) | 3.774508 | 2.26823 | 0.600934 | 0.062 |
| hsa-miR-518e-5p | MIMAT0005450 | chr19:53729838-53729925(+) | 3.882857 | 2.312642 | 0.595603 | 0.239 |
| hsa-miR-519b-5p | MIMAT0005454 | chr19:53695213-53695293(+) | 3.882857 | 2.312642 | 0.595603 | 0.239 |
| hsa-miR-511-5p | MIMAT0002808 | chr10:17845107-17845193(+) | 3.323248 | 1.338412 | 0.402742 | 0.203 |
| hsa-miR-483-5p | MIMAT0004761 | chr11:2134134-2134209(-) | 4.35082 | 1.597655 | 0.367208 | 0.084 |

**Table S12. Effects of confounding variables on miRNA expression in total fraction**

| **Variables** | **miR-217-5p** | **miR-372-3p** | **miR-33a-5p** | **miR-32-5p** | **miR-431-5p** | **miR-30e-5p** | **miR-205-5p** | **miR-193a-5p** | **miR-223-3p** |
| --- | --- | --- | --- | --- | --- | --- | --- | --- | --- |
| Age | r=-0.16  *p*=0.39 | r=-0.16  *p*=0.39 | r=-0.26  *p*=0.16 | r=-0.21  *p*=0.26 | r=0.32  *p*=0.08 | r=-0.32  *p*=0.08 | r=0.13  *p*=0.48 | r=0.5  *p*=.005 | r=-0.16  *p*=0.41 |
| PMI | r=0.09  *p*=0.65 | r=-0.12  *p*=0.52 | r=0.26  *p*=0.15 | r=0.12  *p*=0.51 | r=0.20  *p*=0.27 | r=0.10  *p*=0.59 | r=-0.04  *p*=0.83 | r=-0.09  *p*=0.62 | r=-0.13  *p*=0.49 |
| Brain pH | r=0.18  *p*=.33 | r=0.14  *p*=0.45 | r=-0.04  *p*=0.85 | r=-0.004  *p*=0.98 | r=-0.12  *p*=0.51 | r=0.11  *p*=0.54 | r=-0.35  *p*=0.05 | r=-0.01  *p*=0.95 | r=0.17  *p*=0.38 |
| Sex | t=0.31  df=28  *p*=0.75 | t=1.2  df=28  *p*=0.22 | t=0.86  df=28  *p*=0.39 | t=0.79  df=28  *p*=0.43 | t=0.68  df=28  *p*=0.49 | t=0.69  df=28  *p*=0.49 | t=0.67  df=28  *p*=0.51 | t=1.0  df=28  *p*=0.29 | t=0.66  df=28  *p*=0.52 |
| Alcohol Abuse | t=0.19  df=13  *p*=0.85 | t=2.26  df=13  *p*=0.04 | t=1.38  df=13  *p*=0.19 | t=0.77  df=13  *p*=0.45 | t=2.03  df=13  *p*=0.06 | t=2.08  df=13  *p*=0.06 | t=0.46  df=13  *p*=0.65 | t=0.04  df=13  *p*=0.69 | t=0.08  df=13  *p*=0.93 |
| Antidepressant | t=0.92  df=13  *p*=0.37 | t=1.15  df=13  *p*=0.27 | t=1.14  df=13  *p*=0.27 | t=0.24  df=13  *p*=0.80 | t=0.96  df=13  *p*=0.35 | t=0.52  df=13  *p*=0.61 | t=1.5  df=13  *p*=0.15 | t=0.31  df=13  *p*=0.76 | t=0.49  df=13  *p*=0.62 |

| **Variables** | **miR-376a-3p** | **miR-374a-3p** | **miR-455-3p** | **miR-487a-3p** | **miR-136-3p** | **miR-181c-5p** | **miR-376c-3p** | **miR-629-5p** | **miR-324-5p** |
| --- | --- | --- | --- | --- | --- | --- | --- | --- | --- |
| Age | r=-0.24  *p*=0.20 | r=-0.22  *p*=0.23 | r=0.33  *p*=0.07 | r=-0.38  *p*=0.04 | r=-0.45  *p*=0.01 | r=0.35  *p*=0.06 | r=0.47  *p*=0.008 | r=0.04  *p*=0.84 | r=-0.3  *p*=0.10 |
| PMI | r=0.10  *p*=0.59 | r=0.09  *p*=0.60 | r=-0.18  *p*=0.35 | r=-0.01  *p*=0.94 | r=0.11  *p*=0.55 | r=0.19  *p*=0.31 | r=0.02  *p*=0.90 | r=0.03  *p*=0.89 | r=0.15  *p*=0.43 |
| Brain pH | r=-0.26  *p*=0.16 | r=-0.06  *p*=0.75 | r=-0.003  *p*=0.99 | r=0.06  *p*=0.74 | r=0.15  *p*=0.41 | r=-0.08  *p*=0.68 | r=0.99  *p*=0.60 | r=-0.12  *p*=0.51 | r=0.004  *p*=0.98 |
| Sex | t=0.81  df=28  *p*=0.42 | t=0.81  df=28  *p*=0.43 | t=1.04  df=28  *p*=0.30 | t=0.19  df=28  *p*=0.85 | t=0.10  df=28  *p*=0.92 | t=0.59  df=28  *p*=0.56 | t=0.57  df=28  *p*=0.57 | t=0.16  df=28  *p*=0.88 | t=0.83  df=28  *p*=0.41 |
| Alcohol Abuse | t=0.31  df=13  *p*=0.76 | t=1.0  df=13  *p*=0.311 | t=1.03  df=13  *p*=0.32 | t=0.98  df=13  *p*=0.34 | t=0.76  df=13  *p*=0.45 | t=1.46  df=13  *p*=0.16 | t=0.70  df=13  *p*=0.49 | t=0.005  df=13  *p*=0.99 | t=0.35  df=13  *p*=0.73 |
| Antidepressant | t=1.64  df=13  *p*=0.12 | t=1.38  df=13  *p*=0.18 | t=0.09  df=13  *p*=0.92 | t=0.62  df=13  *p*=0.54 | t=0.38  df=13  *p*=0.71 | t=0.22  df=13  *p*=0.83 | t=2.52  df=13  *p*=0.02 | t=0.77  df=13  *p*=0.45 | t=1.12  df=13  *p*=0.29 |

**Table S13. Effects of confounding variables on miRNA expression in the synaptic fraction**

| **Variables** | **miR-215-5p** | **miR-192-5p** | **miR-202-5p** | **miR-511-5p** | **miR-19b-3p** | **miR-423-5p** | **miR-483-5p** | **miR-219a-2-3p** |
| --- | --- | --- | --- | --- | --- | --- | --- | --- |
| Age | r=0.19  *p*=0.30 | r=0.21  *p*=0.27 | r=-0.21  *p*=0.25 | r=0.14  *p*=0.46 | r=-0.36  *p*=0.048 | r=-0.005  *p*=0.98 | r=0.23  *p*=0.22 | r=-0.061  *p*=0.75 |
| PMI | r=-0.20  *p*=0.20 | r=-0.19  *p*=0.31 | r=0.12  *p*=0.51 | r=-0.16  *p*=0.38 | r=-0.01  *p*=0.94 | r=-0.28  *p*=0.12 | r=-0.23  *p*=0.21 | r=-0.09  *p*=0.60 |
| Brain pH | r=-0.04  *p*=0.80 | r=-0.05  *p*=0.79 | r=0.02  *p*=0.91 | r=-0.001  *p*=0.99 | r=0.09  *p*=0.64 | r=0.31  *p*=0.09 | r=0.04  *p*=0.83 | r=-0.3  *p*=0.88 |
| Sex | t=0.49  df=28  *p*= | t=0.6  df=28  *p*=0.55 | t=0.95  df=28  *p*=0.35 | t=1.23  df=28  *p*=0.23 | t=0.04  df=28  *p*=0.96 | t=0.38  df=28  *p*=0.70 | t=1.7  df=28  *p*=0.09 | t=0.34  df=28  *p*=0.74 |
| Alcohol Abuse | t=0.68  df=13  *p*=0.50 | t=0.53  df=13  *p*=0.60 | t=2.24  df=13  *p*=.04 | t=1.87  df=13  *p*=0.08 | t=2.14  df=13  *p*=0.05 | t=0.95  df=13  *p*=0.36 | t=0.67  df=13  *p*=0.52 | t=2.10  df=13  *p*=0.06 |
| Antidepressant | t=0.36  df=13  *p*=0.72 | t=0.52  df=13  *p*=0.61 | t=1.25  df=13  *p*=0.23 | t=0.69  df=13  *p*=0.50 | t=1.89  df=13  *p*=0.08 | t=0.78  df=13  *p*=0.44 | t=0.63  df=13  *p*=0.53 | t=0.69  df=13  *p*=0.50 |

**Table S14. Predicted targets based on 14 uniquely expressed miRNAs in synaptosome (expression change > 20%)**

| **Gene symbol** | **Candidate miRNAs** |
| --- | --- |
| TGFβ | miR-202-5p, miR-519d-3p |
| CREBBP | miR-2276-3p |
| LIMK1 | miR-519d-3p |
| GRIN3A | miR-520a-3p |
| CACNA1A | miR-449c-5p |
| NOTCH1 | miR-449c-5p |
| PPP3CA | miR-512-3p, miR-519d-3p |
| WNT5A | miR-512-3p |
| LEF1 | miR-512-3p, miR-520a-3p |
| CAMK2 | miR-519d-3p, miR-520a-3p |
| MAP3K2 | miR-19a-3p, miR-512-3p |


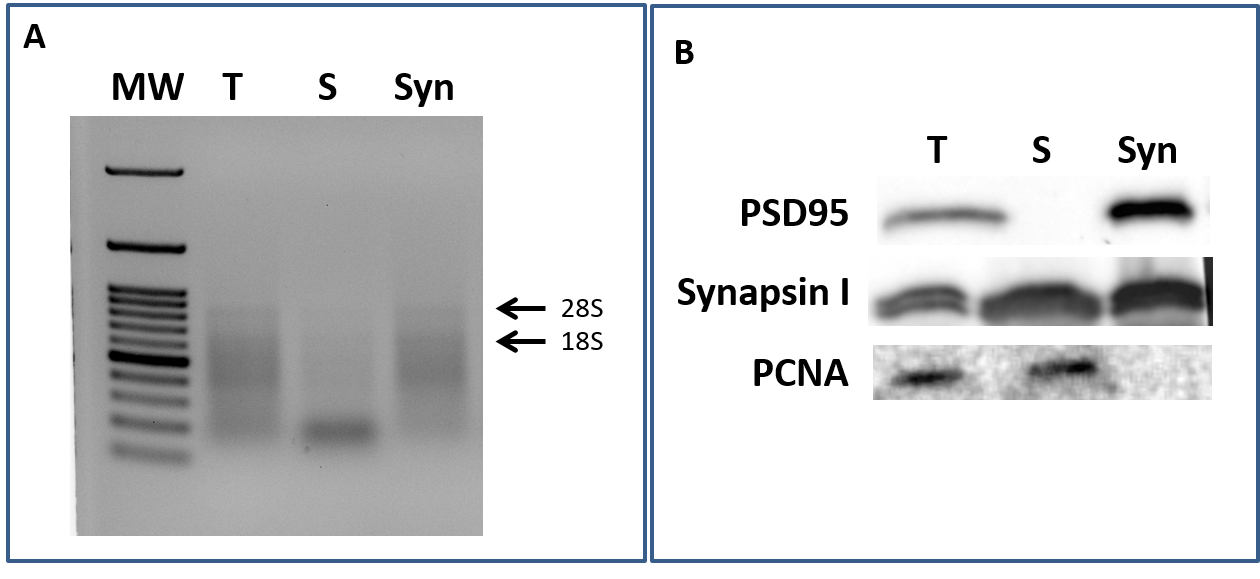


**Figure S1.** **Validation of RNA quality with gel electrophoresis and purity of synaptosomes with Western Blot.**

A) An equal amount of RNAs (500ng) was loaded on to 0.8% TAE Agarose gel and stained with ethidium bromide. MW is 100bp plus ladder size marker.

B) An equal amount of protein (20µg) was loaded and subject to PAGE.

T, total fraction; S, S fraction (20,000 x 20 min supernatant); Syn, synaptosome


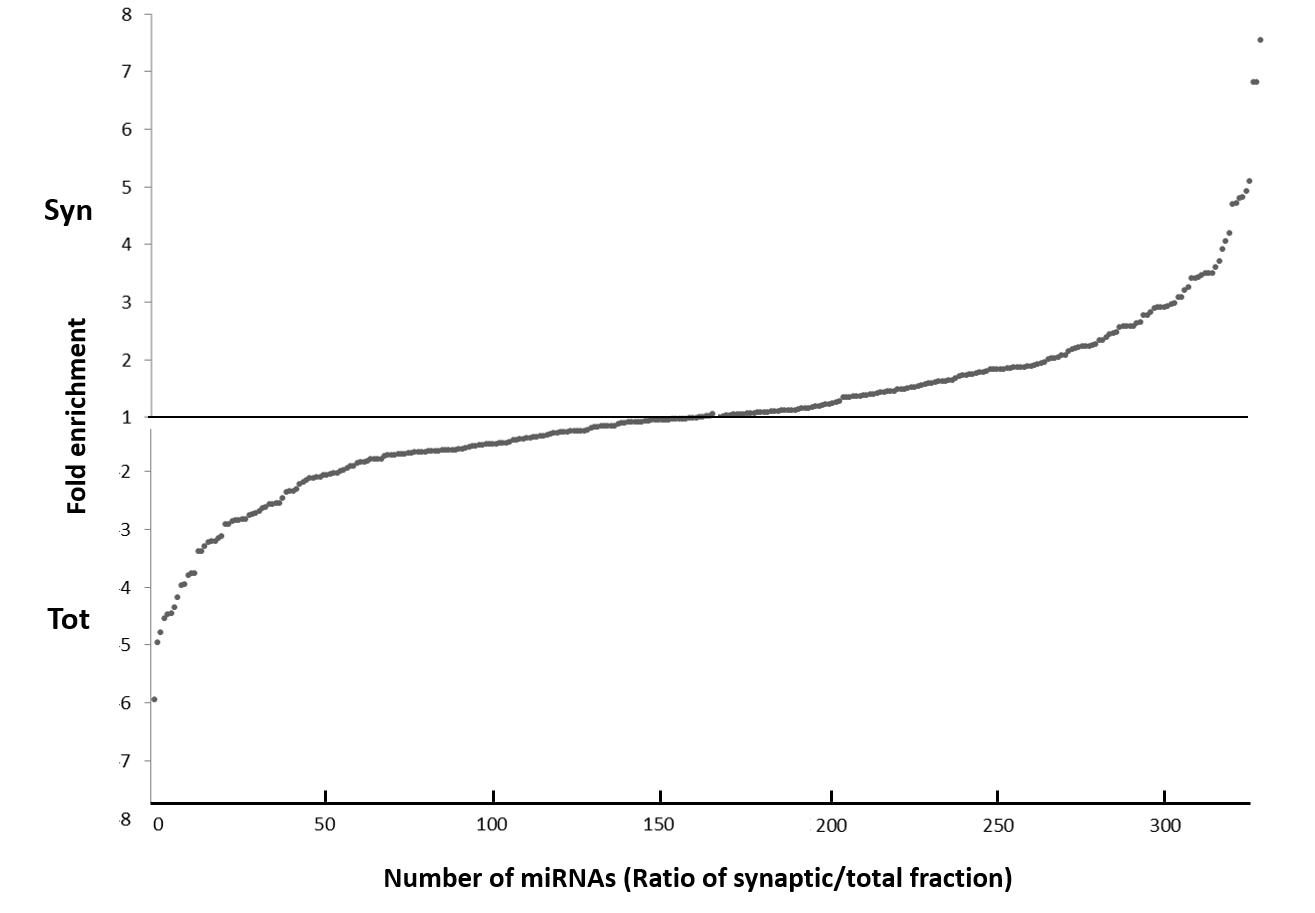


**Figure S2.** **Enrichment ratios of synaptosome miRNAs compared to total fraction miRNAs.** miRNA expressions ratios of synaptosome/total fraction in control subjects was analyzed using miRNA-seq data. Syn, synaptosome; Tot, total fraction.

**
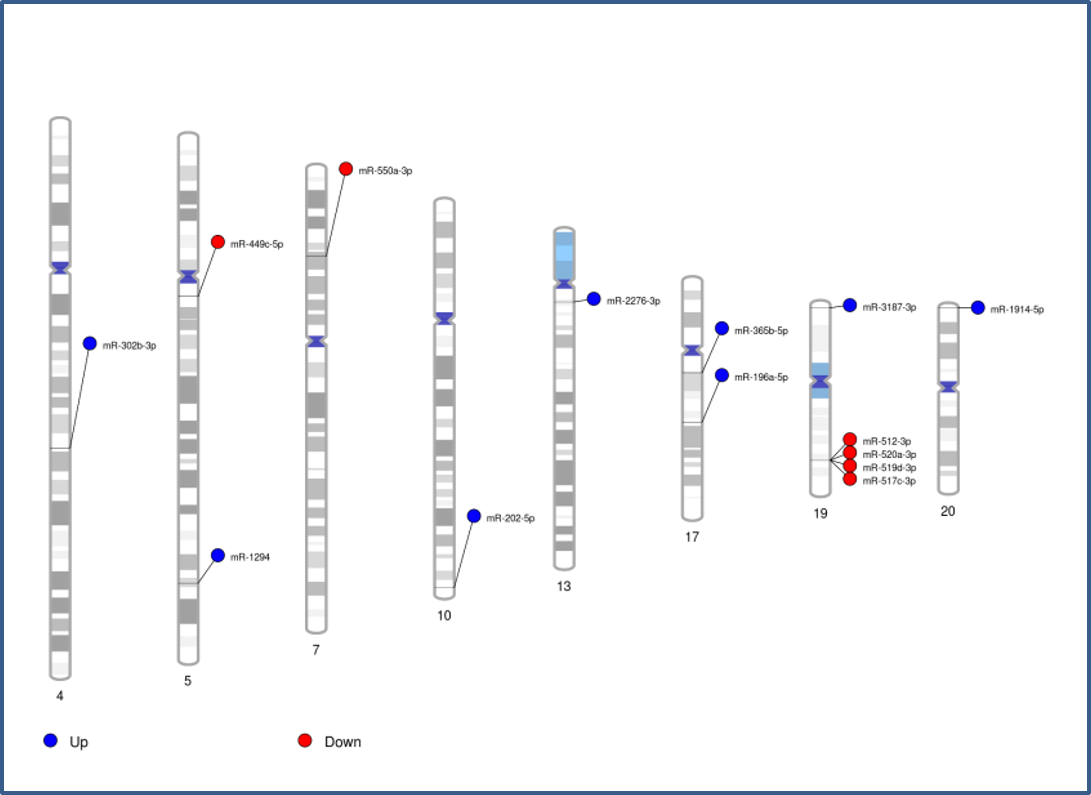
**

**Figure S3.** **Phenogram showing chromosomal localization of miRNAs uniquely expressed in synaptosomes**. Relative localization of uniquely expressed synaptic miRNAs (with 20% change) on different chromosomes. Blue (up) and red (down) colors show miRNAs found up and downregulated respectively.


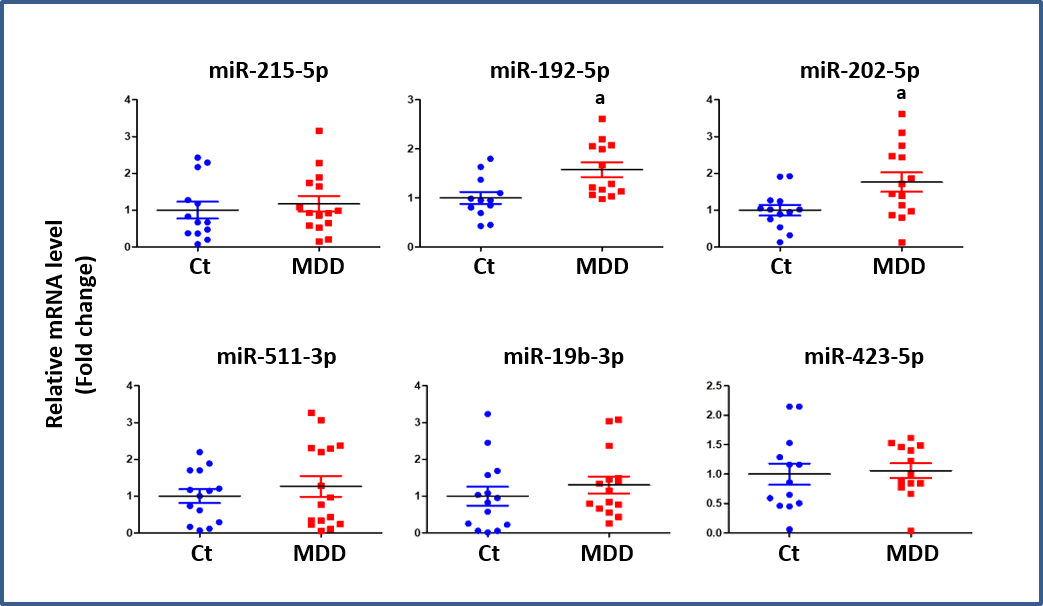


**Figure S4.** **mRNA expression of miRNA biogenesis genes in synaptosomes.** DROSHA: df = 20; t = -1.461, p = 0.160; DICER1: df = 26; t = -0.467, p = 0.644; TARBP2: df = 27; t = -1.058, p = 0.300; AGO2: df = 24; t = -0.490, p = 0.961. Geometric mean (GAPDH, ACTB, and ribosomal 18S RNA) was used as normalizer. Values denote average ± SEM. ‘a’ denotes the statistical significance between control and MDD subjects. Ct, control; miRNA, microRNA; MDD, major depressive disorder.

**Supplemental References**

1 Zalcman S EJ. Diagnostic Evaluation After Death. NIMH Nueroscienes Research Branch, Department of Research Assessment and Training, New York State Psychiatryc Institution. 1983.

2 Spitzer RL, Williams JB, Gibbon M, First MB. The Structured Clinical Interview for DSM-III-R (SCID). I: History, rationale, and description. Arch Gen Psychiatry. 1992;49(8):624-9.

3 First MS, RL.; Gibbon, M.; Williams, JBW. Structured Clinical Interview for DSM-IV-TR Axis I Disorders, Research Version*.* Non-patient ed. Biometric Research, New York State Psychiatric Institute: New York, NY; 2002.

4 Smalheiser NR, Collins BJ. Coordinate enrichment of cranin (dystroglycan) subunits in synaptic membranes of sheep brain. Brain Res. 2000;887(2):469-71.

5 Lugli G, Larson J, Martone ME, Jones Y, Smalheiser NR. Dicer and eIF2c are enriched at postsynaptic densities in adult mouse brain and are modified by neuronal activity in a calpain-dependent manner. J Neurochem. 2005;94(4):896-905.

6 Roy B, Dunbar M, Shelton RC, Dwivedi Y. Identification of MicroRNA-124-3p as a Putative Epigenetic Signature of Major Depressive Disorder. Neuropsychopharmacology. 2017;42(4):864-75.

7 Langmead B. Aligning short sequencing reads with Bowtie. Curr Protoc Bioinformatics. 2010;Chapter 11:Unit 11 7.

8 Robinson MD, Oshlack A. A scaling normalization method for differential expression analysis of RNA-seq data. Genome Biol. 2010;11(3):R25.

9 Robinson MD, McCarthy DJ, Smyth GK. edgeR: a Bioconductor package for differential expression analysis of digital gene expression data. Bioinformatics. 2010;26(1):139-40.

10 Livak KJ, Schmittgen TD. Analysis of relative gene expression data using real-time quantitative PCR and the 2(-Delta Delta C(T)) Method. Methods. 2001;25(4):402-8.
